# Feeder cell training shapes the phenotype and function of in vitro expanded natural killer cells

**DOI:** 10.1002/mco2.740

**Published:** 2024-09-23

**Authors:** Fei Gao, Mauricio Campos Mora, Michael Constantinides, Loïs Coënon, Caroline Multrier, Loïc Vaillant, Julien Peyroux, Tianxiang Zhang, Martin Villalba

**Affiliations:** ^1^ IRMB University of Montpellier INSERM CHR Montpellier Montpellier France; ^2^ Department of Pathology School of Basic Medicine Central South University Changsha China; ^3^ Department of Immunobiology Yale University School of Medicine New Haven Connecticut USA; ^4^ Institut du Cancer Avignon‐Provence Sainte Catherine Avignon France; ^5^ IRMB Univ Montpellier INSERM CHU Montpellier CNRS Montpellier France

**Keywords:** cytokine‐producing, cytotoxicity, expanded conventional NK (ecNK) cells, expanded FcRγ^–^ NK (eg‐NK) cells, expanded NK (eNK) cells, feeder cells

## Abstract

Natural killer (NK) cells are candidates for adoptive cell therapy, and the protocols for their activation and expansion profoundly influence their function and fate. The complexity of NK cell origin and feeder cell cues impacts the heterogeneity of expanded NK (eNK) cells. To explore this, we compared the phenotype and function of peripheral blood‐derived NK (PB‐NK) and umbilical cord blood‐derived NK (UCB‐NK) cells activated by common feeder cell lines, including K562, PLH, and 221.AEH. After first encounter, most PB‐NK cells showed degranulation independently of cytokines production. Meanwhile, most UCB‐NK cells did both. We observed that each feeder cell line uniquely influenced the activation, expansion, and ultimate fate of PB eNK and UCB eNK cells, determining whether they became cytokine producers or killer cells. In addition, they also affected the functional performance of NK cell subsets after expansion, that is, expanded conventional NK (ecNK) and expanded FcRγ^–^ NK (eg‐NK) cells. Hence, the regulation of eNK cell function largely depends on the NK cell source and the chosen expansion system. These results underscore the significance of selecting feeder cells for NK cell expansion from various sources, notably for customized adoptive cell therapies to yield cytokine‐producing or cytotoxic eNK cells.

## Graphical abstract

NK cells from PB and UCB represent two heterogeneous populations, encompassing cNK and g‐NK cells. The ultimate functionality of expanded NK (eNK) cells hinges primarily on the source of NK cells and the stimulatory signals they receive during the expansion process, for example, stimulation of feeder cells. Notably, cytokine production and cytotoxicity are mostly independent and partially related.

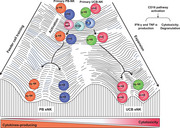

## 1 INTRODUCTION

Adoptive transfer of natural killer (NK) cell therapy is currently undergoing a blooming development, notably encouraged by the achievements of chimeric antigen receptor (CAR)‐NK in the treatment of hematologic malignancies.^1^, ^2^, ^3^ NK cells possess the ability to rapidly eliminate abnormal cells experiencing malignant transformation and viral infection.^4^ They play a crucial role in cytotoxicity, cytokine secretion, and immune coordination during the process of immune surveillance.^5^ Their cytotoxic activity is mediated by the release of perforin and granzyme B (Gzm B), resulting in the apoptosis and lysis of target cells. Additionally, NK cells can produce various cytokines and chemokines, such as interferon (IFN)‐γ and tumor necrosis factor (TNF)‐α, interleukins (e.g., IL‐1α, IL‐1β, IL‐2, IL‐8, IL‐22, and IL‐10), granulocyte‐macrophage colony‐stimulating factor, leukemia inhibitory factor, chemokine ligand 5, and X‐C motif chemokine ligand 1, all of which contribute to various immunomodulatory function.^6^ The production and mechanism of action of cytokines and cytotoxicity differ significantly,^7^, ^8^, ^9^ leading to an incomplete synchronization between cytokines production and the manifestation of cytotoxicity.

Owing to their long‐term coevolution with chronic viruses, certain NK cell phenotype and function exhibit a spectrum of diversity, encompassing epigenetic modification.^10^ Recent studies have unveiled the functional and phenotypic diversities of NK cells that were not appreciated before. For example, a subset of human NK cells, termed adaptive NK or FcRγ^–^ NK (g‐NK) cells, exhibits enhanced antibody‐dependent cell‐mediated cytotoxicity (ADCC), clonal‐like expansion, and long‐term survival, which are classical adaptive immune features.^11^, ^12^, ^13^, ^14^ They lack the signaling adapter FcRγ chain but express abundant CD3ζ chain, partially overlapping with NKG2C^+^ NK cells.^13^, ^15^ The deficiency in FcRγ and the high expression of NKG2C are sometimes independent phenomena.^16^ g‐NK cells were initially identified in peripheral blood (PB). They account for up to 85% of circulating NK cells, and in some, they even surpass the number of circulating memory CD8^+^ T cells.^17^ g‐NK cells express low levels of NKp46 and NKp30, and compared with conventional NK (cNK or FcRγ^+^ NK) cells, they show poorer reactivity to K562 cells, indicating their weaker natural killing ability. However, g‐NK cells demonstrate an enhanced response to CD16 cross‐linking.^11^ This subset is strongly linked to human cytomegalovirus (HCMV) infection and its reactivation after organ transplantation.^15^, ^18^, ^19^, ^20^ HCMV is a widely existing β‐herpesvirus. After the initial infection, HCMV can establish a latent state and persist for life in the body.^21^ Over 80% of individuals in the world are latently infected with HCMV, and in some countries, the seropositive rate of HCMV is as high as 96%.^22^, ^23^ Normally, HCMV can be transmitted to infants through congenital and perinatal infection, or through breastfeeding.^24^, ^25^, ^26^ In this context, the g‐NK subset sourced from umbilical cord blood (UCB) is also present and characterized by the defect in FcRγ, also namely UCB g‐NK. However, unlike g‐NK cells sourced from PB (PB g‐NK), UCB g‐NK cells do not possess the enhanced CD16 responsiveness in an antibody‐dependent manner.^27^

NK cell activation hinges on the disruption of the equilibrium between activating and inhibitory receptors.^28^ Typically, quiescent NK cells require a two‐stage activation process consisting of a first encounter followed by triggering. The successful lysis of NK‐sensitive tumor cells involves the sequential completion of these two steps, with resistance to killing often emerging during the encountering phase.^29^ After encountering tumor cells or undergoing cytokine‐mediated preactivation, NK cells experience changes in their functional and transcriptional characteristics. These include elevated levels of secreted cytokines or increased expression of molecules associated with cytotoxicity.^30^ These principles also can be applied to protocols for NK cell expansion, where NK cell origin and in vitro stimuli emerge as crucial factors.^31^ Commonly used NK cell sources include UCB, PB, NK cell lines, and induced pluripotent stem cells.^32^ The expansion process generally involves the utilization of feeder cells, such as autologous accessory cells, engineered cells (e.g., K562 expressing NK activating ligands), and Epstein–Barr virus‐immortalized lymphoblastoid cell lines (EBV‐LCLs).^33^, ^34^, ^35^ Different feeder cells activate and expand NK cells through various pathways, such as overexpressing membrane‐bound IL‐15 (mbIL‐15), CD137L, OX40L, mbIL‐18, and mbIL‐21, as well as hybrid HLA‐E molecules.^33^, ^36^, ^37^ Despite this, their influence on the function and activity of expanded NK (eNK) cells through sustained stimulation or training remains uncertain. The cell lines K562 and 721.221, both lacking HLA and highly responsive to NK cell‐mediated cytotoxicity, have been widely employed.^38^ HLA‐E can induce the activation and expansion of NKG2C^+^ NK cells by presenting peptides.^39^, ^40^ Based on the positive correlation between FcRγ^–^ and NKG2C^+^ NK cell subsets, researchers use K562 or 721.221 overexpressing HLA‐E (referred to K562.AEH and 221.AEH, respectively) as feeder cells to expand NK cells, along with the g‐NK subset present therein.^41^, ^42^, ^43^ Yang et al.^38^ found that the use of 721.221 cells overexpressing mbIL‐21 (221‐mIL‐21) leads to improved NK cell expansion, cytotoxicity, transduction potential, stemness, and a phenotype associated with memory‐like NK cells, compared with K562 cells overexpressing mbIL‐21 (K562‐mIL‐21). The EBV‐LCL PLH cell line expresses multiple HLA molecules and effectively expands NK cells from UCB, exhibiting high cytotoxicity; however, the underlying mechanism remains not fully elucidated.^44^, ^45^ The foregoing suggests that various feeder cells may display specificity in activating NK cells, thereby affecting their eventual functional outcome.

To ensure the optimal efficacy of adoptive NK cell therapy, it is crucial to conduct a comprehensive evaluation of the composition and effector function of NK cell subsets, as well as select an appropriate blood source and an efficient in vitro expansion system. Ullrich's team found in 2017 92% of clinical studies choose PB‐derived NK (PB‐NK) cells, with 79% are sourced from healthy donors and 13% from patients.^34^ It has been reported that patients with a higher frequency of g‐NK cells exhibit prolonged overall survival and progression‐free survival after trastuzumab treatment.^46^ Notably, in 25−30% of HCMV^+^ individuals, the PB g‐NK subset only accounts for 3−10% of total NK cells. Given this relatively low proportion, effective in vitro is necessary for g‐NK cell use in clinical research.^17^ However, to date, only Indapta Therapeutics has reported their effective expansion using NK MACS Good Manufacturing Practices (GMP) medium (Miltenyi, Germany), specialized feeder cells, and proinflammatory cytokines including IL‐2; achieving a 4500‐expansion fold after 14 days.^17^ Now days, the stimulatory signals provided by feeder cells and/or the specific components of the medium is mainly unknown. The above‐mentioned group observed that when switching to another GMP‐compliant medium, g‐NK cells expanded in the two different media exhibit differences in ADCC activity, degranulation, and IFN‐γ and TNF‐α production while responding to the stimulation of myeloma cell lines KMS‐34 and LP1 (Miltenyi; Indapta Therapeutics, USA).^17^ Alongside with the clinical use of NK cells, UCB‐derived NK (UCB‐NK) cells expressing anti‐CD19 CAR and IL‐15 display favorable clinical efficacy.^1^ The adoption of CAR‐NK cells derived from cord blood units (CBUs) with nucleated red blood cells count below 8 × 10^7^ and a frozen storage duration shorter than 24 h are served as the most significant predictive indicator for a favorable prognosis.^2^ The optimized CAR‐NK cell treatment group achieve a 1‐year overall survival rate and progression‐free survival rate of 94 and 69%, respectively, whereas the nonoptimized group only achieve 48 and 5%, respectively. These optimal CAR‐NK cells are functionally potent and enriched in effector function‐related genes, such as *NKG2D*, *CD16*, *2B4*, *T‐bet*, *EOMES*, *Perforin*, and *Gzm A*.^2^ Currently, researchers are focusing on exploring the potential of PB g‐NK cells in anti‐tumor therapy. However, whether UCB g‐NK cells possess clinical application value after expansion remains unknown. Evaluating the impact of the functional changes in g‐NK cells after expansion on the overall efficacy of the NK cell product is crucial for preclinical selection.

Herein, we studied the effector function of NK cells expanded with distinct feeder cells and found that NK cells behaved differently after being expanded by distinct systems. PB‐NK cells demonstrated heightened reactivity to 221.AEH cells compared with PLH cells, while UCB‐NK cells exhibited an opposite trend. Notably, when 221.AEH cells were used as feeder cells to expand PB‐NK cells, the functional discrepancy in CD16 pathway response between expanded cNK (ecNK) and expanded g‐NK (eg‐NK) cells, which was observed in primary PB‐NK cells, persisted. PLH‐mediated UCB‐NK expansion resulted in a near loss of cytokines production capability while retaining elevated cytotoxic potential. These findings highlight the necessity of choosing specific NK expansion systems for adoptive therapy accordingly, depending on whether the therapeutic option aims to modulate the tumor microenvironment (TME) through robust cytokines production or prioritizes tumor eradication with high cytotoxicity.

## 2 RESULTS

### 2.1 Feeder cells elicit unique signals for NK cell activation

**FIGURE 1:**
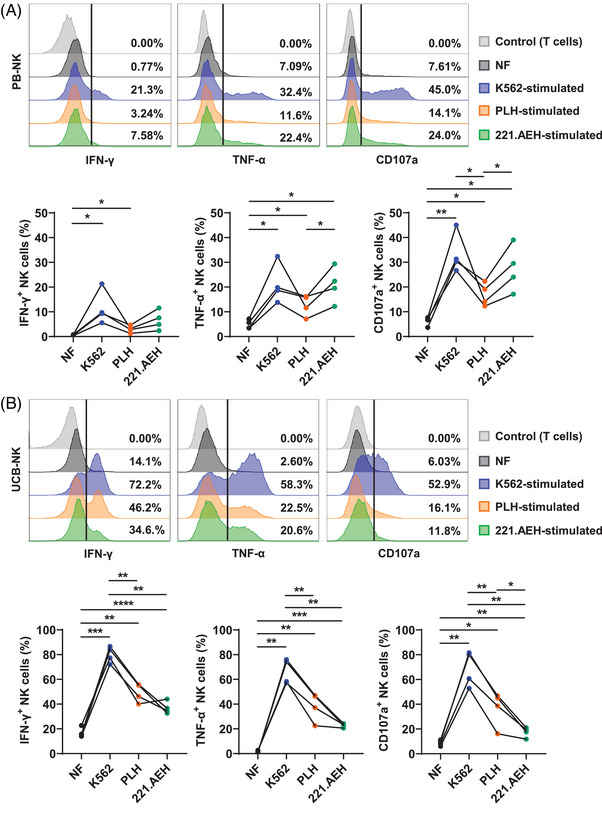
Different feeder cells deliver diverse activation signals to NK cells. (A) Peripheral blood‐derived NK (PB‐NK) cells were cultured with three types of feeder cells (K562, PLH, and 221.AEH) for 6 h. The production of IFN‐γ and TNF‐α, as well as CD107a expression, was assessed by flow cytometry (FCM). Representative FCM histograms are shown here. Line graphs showing the proportion of IFN‐γ^+^, TNF‐α^+^, and CD107a^+^ subsets in PB‐NK cells (*n* = 4). The no feeder cell (NF) condition served as basal expression. In this condition, NK cells were cultured in the medium containing 100 IU/mL IL‐2 and 5 ng/mL IL‐15, but no feeder cells. (B) Umbilical cord blood‐derived NK (UCB‐NK) cells were treated and analyzed as described in graph (A) (*n* = 4). Dots linked by a line represent data collected from the same donor. Two‐tailed paired *t*‐tests were used for all comparisons. *p < 0.05; **p < 0.01; ***p < 0.001; ****p < 0.0001.

We assessed PB‐NK cell responsiveness to stimulation using various feeder cells, that is, K562, PLH, and 221.AEH, by evaluating cytokines production (IFN‐γ and TNF‐α) and degranulation (CD107a) (Figures 1A and S1A). All these parameters exhibited elevated levels when PB‐NK cells encountered K562 cells, with an intermediate effect observed for 221.AEH cells and the lowest effect for PLH cells (Figure 1A). Upon further analysis of the mean fluorescence intensity among the positive population, it was unequivocally confirmed that the interaction with K562 cells elicited the highest response (Figure S1A). Subsequently, UCB‐NK cells were stimulated, and once again, K562 cells demonstrated the highest efficacy in stimulating NK cells compared with other feeder cells. In contrast, PLH cells were more efficient than 221.AEH cells in triggering degranulation and TNF‐α expression in UCB‐NK cells (Figures 1B and S1B). These observations suggest that different feeder cells impart specific stimulatory signals to PB‐NK and UCB‐NK cells.

To assess whether the cells producing cytokines overlap with those undergoing degranulation, we analyzed cells that express either one, two, or all three of the markers IFN‐γ, TNF‐α, and CD107a (Table 1). Both no feeder cell (NF)‐stimulated PB‐NK and UCB‐NK cells generally exhibited minimal expression of these markers, with few cells being CD107a^+^. After stimulation, regardless of the type of feeder cells used, the percentage of CD107a^+^ PB‐NK cells rose significantly. Nevertheless, the majority of these cells remained noncytokine producers (Table 1). A comparatively higher fraction of PB‐NK cells within the CD107a^+^ subset exhibited cytokines production compared with the CD107a^–^ subset (Figure S2A and Tables S1 and S2). This indicates that PB‐NK cells can engage in vitro in cytokines production and degranulation independently. Upon stimulation, a substantial portion of responsive UCB‐NK cells upregulated the expression of all three markers, IFN‐γ, TNF‐α, and CD107a, or at least two of them (Table 1). Moreover, a considerable fraction of UCB‐NK cells within the CD107a^+^ subset exhibited expression of either one or both cytokines. It is noteworthy that a few degranulated cells did not produce cytokines (Figure S2B and Table S1). These findings suggest a closer association between cytokines production and degranulation in UCB‐NK cells compared with PB‐NK cells following target cell encounter.

**TABLE 1:** The ability of PB‐NK and UCB‐NK cells to produce IFN‐γ and TNF‐α, as well as CD107a expression, was assessed under different feeder cell stimulation conditions.

Cytokines			% in PB‐NK cells				% in UCB‐NK cells			
I	T	C	NF	K562	PLH	221.AEH	NF	K562	PLH	221.AEH
+	+	+	0.25 ± 0.14	7.35 ± 4.72	1.22 ± 0.50	2.87 ± 1.71	0.46 ± 0.60	35.2 ± 13.2	19.9 ± 12.9	7.18 ± 3.47
+	+	–	0.12 ± 0.10	1.15 ± 0.48	0.79 ± 0.41	1.72 ± 1.05	0.13 ± 0.14	8.35 ± 4.98	2.87 ± 1.71	4.21 ± 1.88
+	–	+	0.14 ± 0.12	2.50 ± 1.82	0.55 ± 0.33	1.21 ± 0.90	0.90 ± 0.47	3.80 ± 1.84	0.70 ± 0.35	0.71 ± 0.72
–	+	+	0.71 ± 0.11	4.92 ± 1.31	2.90 ± 1.08	5.97 ± 2.76	0.31 ± 0.07	9.40 ± 3.65	4.65 ± 3.05	2.00 ± 0.80
+	–	–	0.16 ± 0.10	0.65 ± 0.23	0.52 ± 0.24	0.89 ± 0.46	0.20 ± 0.16	1.60 ± 0.92	0.70 ± 0.33	1.56 ± 0.90
–	+	–	0.71 ± 0.28	2.60 ± 0.98	2.97 ± 0.96	4.48 ± 1.66	0.76 ± 0.76	5.44 ± 2.04	6.73 ± 3.17	9.38 ± 3.56
–	–	+	5.27 ± 1.62	18.6 ± 2.83	12.3 ± 3.95	17.4 ± 6.47	1.67 ± 0.42	7.46 ± 4.42	2.77 ± 1.82	1.78 ± 1.39
–	–	–	92.5 ± 1.73	61.7 ± 8.20	78.2 ± 4.89	64.8 ± 10.3	93.7 ± 0.95	19.2 ± 10.9	46.4 ± 15.1	67.9 ± 2.63

Analysis of cytokines production after coculturing CD56^+^CD3^–^ NK cells with K562, PLH, or 221.AEH cells for 6 h. The negative control group, denoted as “no feeder cells (NF)” was included. The median percentage of cytokines production was assessed by FCM. The total percentage of cell proportion, from gating G1 to G8 (from top to bottom), is approximately 100%. Sample numbers: PB‐NK *n* = 4; UCB‐NK *n* = 5. ± represent SD.

Abbreviations: FCM, flow cytometry; G, group; I, IFN‐γ; NK, natural killer; PB, peripheral blood; SD, standard deviation; T, TNF‐α; UCB, umbilical cord blood.

**FIGURE 2:**
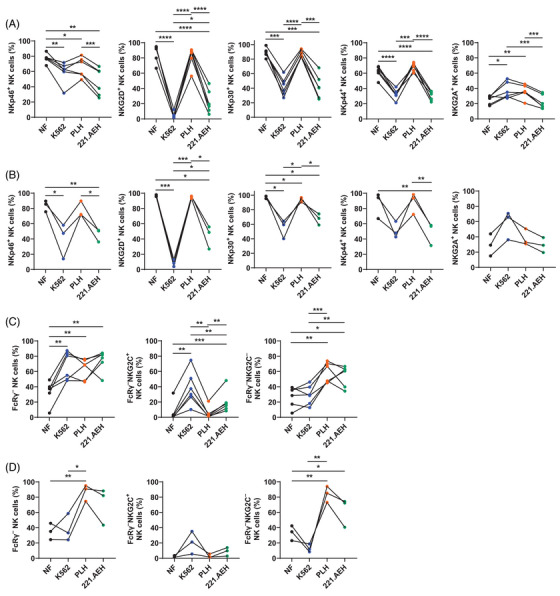
Changes in NK cell phenotype after encountering feeder cells. (A and B), Line graphs showing the expression of NKp46, NKG2D, NKp30, NKp44, and NKG2A on PB‐NK (A) and UCB‐NK (B) cells after 3‐day stimulation using K562, PLH, and 221.AEH cells (PB‐NK, *n* = 6; UCB‐NK, *n* = 3). (C and D) Primary NK cells were cocultured with three feeder cells (i.e., K562, PLH, and 221.AEH) for 3 days. Line graphs depicting the proportion of FcRγ^–^ and NKG2C^+/–^ cells within PB‐NK (C) and UCB‐NK (D) cells (PB‐NK, *n* = 6; UCB‐NK, *n* = 3). Dots linked by a line represent data collected from the same donor. Two‐tailed paired *t*‐tests were used for all comparisons. *p < 0.05; **p < 0.01; ***p < 0.001; ****p < 0.0001.

We next evaluated alterations in the NK cell phenotype induced by these feeder cells 3 days after the initial encounter (Figures 2A,B and S3A). This time point was chosen to allow for changes in NK cell characteristics and to stabilize the expression of the inhibitory and/or activating receptors. PLH cells consistently maintained expression of activating receptors expressed on PB‐NK cell membrane, including NKp46, NKG2D, NKp30, and NKp44. In contrast, PB‐NK cells stimulated by 221.AEH and K562 cells exhibited reduced expression of these receptors. 221.AEH cells outperformed K562 cells in maintaining NKG2D expression. Compared with the NF group, both K562 and PLH cells induced upregulation of the inhibitory receptor NKG2A expressed on PB‐NK cells (Figures 2A and S3A). Although only three samples of UCB‐NK cells were stimulated by various factors including NF, K562, PLH, and 221.AEH, the results consistently demonstrated a similar trend in the surface expression of NKp46, NKG2D, NKp30, and NKp44 to that of PB‐NK cells. Specifically, NF and PLH stimulation maintained high expression of these activating receptors, while K562 and 221.AEH stimulation promoted downregulation of their expression, with the exception of the lack of an increase in NKG2A expression (Figures 2B and S3A). The above results suggest that feeder cells affect these markers independently of the source of NK cells.

To investigate whether different NK cell subsets are differentially affected by the feeder cells, we examined the impact of a 3‐day stimulation with various feeder cells on the proportion of the g‐NK subset (Figures 2C,D and S3B). Stimulation with all feeder cells, that is, K562, PLH, and 221.AEH, equally increased the proportion of the PB g‐NK subset (Figures 2C and S3B). K562 cells preferentially elevated the proportion of the NKG2C^+^ NK subset, whereas PLH cells enhanced the proportion of the NKG2C^–^ NK subset among PB g‐NK cells. In contrast to the NF group, 221.AEH cells not only promoted an increase in the proportion of the NKG2C^+^ NK subset but also facilitated an increase in the proportion of the NKG2C^–^ NK subset (Figures 2C and S3B). Upon stimulation with PLH cells, the UCB g‐NK subset's proportion was increased compared with the NF group, but remained unchanged after exposure to K562 and 221.AEH cells. This increase was primarily evident in the NKG2C^–^ NK subset (Figures 2D and S3B). The above findings suggest that the choice of feeder cells may have an impact on the composition and enrichment of NK cell subsets during expansion.

### 2.2 Bulk NK and g‐NK subsets are expanded in primary PB‐NK cells with 221.AEH feeder cells

g‐NK cells, particularly those from PB not UCB, represent a subset exhibiting enhanced cytokines production following CD16 pathway activation.^27^ Preliminary findings indicated that K562.AEH cells expanded PB‐NK cells at least similarly to K562 cells (Figure S4A,B). Furthermore, PB eNK cells expanded with K562.AEH cells displayed a higher proportion of the eg‐NK subset, characterized by the absence of NKp30, as well as an increased representation of the NKG2C^+^ subset (Figure S4C–E). Despite this, the capacity of K562.AEH as feeder cells for expanding PB‐NK cells was constrained, with an expansion fold of less than 20 times (Figure S4B). In agreement with Yang et al.,^38^ 221.AEH cells induced higher expansion efficiency of PB‐NK cells that could achieve a maximum of 106‐fold (Figure S4F).

To evaluate the difference in the ability of PLH and 221.AEH cells as feeder cells in expanding PB‐NK and UCB‐NK cells, we examined their proliferation status after 7 days of stimulation with the feeder cells (Figure S5). PLH cells did not enhance the expansion of PB‐NK cells or increase the proportion of the PB eg‐NK subset upon stimulated with cytokines (IL‐2+IL‐15) (Figure S5A,B). In contrast, 221.AEH cells exhibited superior stimulatory properties for NK cells, particularly those originating from the PB source, including promoting the proliferation of PB eNK cells and enriching the g‐NK (CTV^low^FcRγ^–^) subset (Figure S5A,B). PLH cells effectively expanded UCB‐NK cells compared with PB‐NK cells, though their stimulatory effect was less pronounced than that of 221.AEH cells (Figure S5A). Furthermore, PLH as feeder cells could enrich the NKG2C^+^ subset in UCB eNK cells (Figure S5C).

**FIGURE 3:**
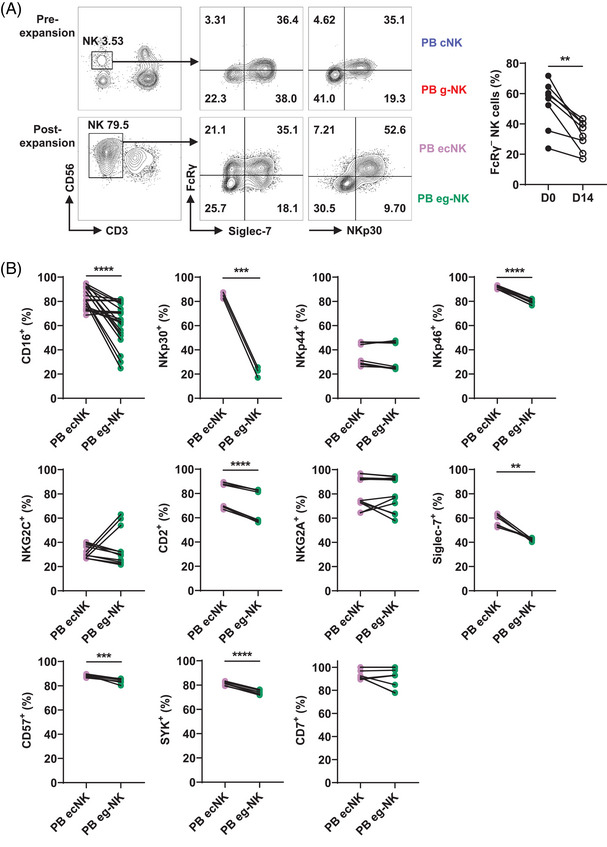
Expansion of bulk NK and g‐NK subsets in PB‐NK cells with 221.AEH feeder cells. (A) PB‐NK were expanded using 221.AEH as feeder cells for 14 days, and the phenotype of expanded conventional NK (ecNK) or expanded FcRγ^–^ NK (eg‐NK) cells was subsequently analyzed. The left graph, FCM contour plots, showing a representative donor's PB‐NK cells pre‐ and postexpansion. While the right graph illustrating the proportion of g‐NK cells within PB pre‐ and postexpansion (*n* = 8). (B) Phenotyping of PB ecNK versus PB eg‐NK cells. Before–after plot graphs representing the percentage of positive cells for each analyzed marker, including CD16 (*n* = 22), NKp30 (*n* = 3), NKp44 (*n* = 8), NKp46 (*n* = 8), NKG2C (*n* = 11), CD2 (*n* = 8), NKG2A (*n* = 14), Siglec‐7 (*n* = 6), CD57 (*n* = 8), SYK (*n* = 8), and CD7 (*n* = 14). Dots linked by a line represent data collected from the same donor. Two‐tailed paired *t*‐tests were used for all comparisons. **p < 0.01; ***p < 0.001; ****p < 0.0001.

Therefore, we selected 221.AEH as feeder cells for the expansion of PB‐NK cells and evaluated the phenotypic differences between PB ecNK and PB eg‐NK cells. After expansion, the expression of CD16 by eNK cells remained stable, suggesting their capability for ADCC (Figure S6A). During the expansion process, the percentage of FcRγ^–^ subset decreased across all PB eNK cell batches (Figure 3A). The average percentage of the PB ecNK subset surpassed that of the PB eg‐NK subset, with 55.4 ± 7.46% for PB ecNK and 44.6 ± 7.46% for PB eg‐NK (Figure S6B). Despite this, PB eg‐NK cells exhibited efficient expansion under the 221.AEH feeder cell system when compared with their pre‐expansion numbers, with fold increases ranging from 2 to 54 times (Figure S6C).

Recently, we have described that primary PB cNK cells express higher levels of CD16, NKp30, Siglec‐7, and SYK compared with PB g‐NK cells.^27^ Conversely, the expression of CD57 and NKG2C are lower in PB cNK cells. The expression of NKp44, NKp46, NKG2A, CD2, and CD7 are comparable between these two populations. In the present study, we observed higher expression of CD16, NKp30, NKp46, CD2, Siglec‐7, CD57, and SYK in PB ecNK cells compared with PB eg‐NK cells. However, there were no significant differences in the expression of NKp44, NKG2A, NKG2C, and CD7 (Figure 3B). These results suggest that PB eg‐NK cells undergo some phenotypic changes compared with primary PB g‐NK cells, but they essentially maintain a distinct phenotype from PB ecNK cells.

### 2.3 PB eNK cells inherit the cytokines production characteristic of parental NK cells but show limited cytotoxicity after expansion

**FIGURE 4:**
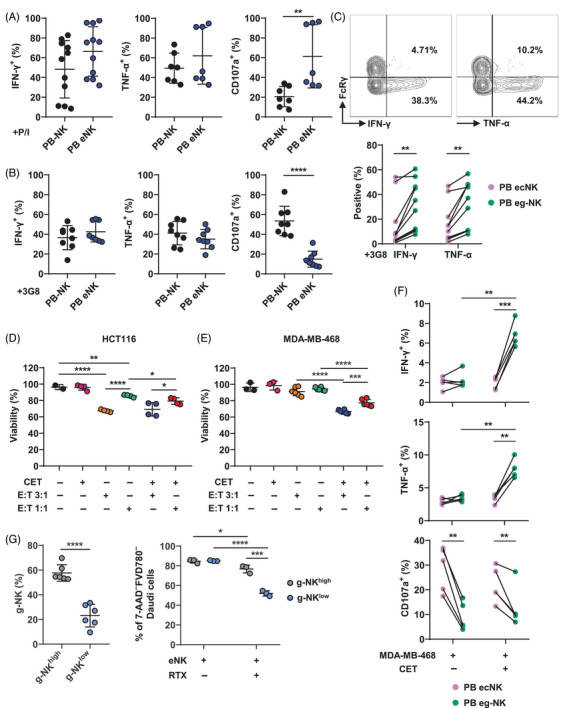
Cytokine production characteristic is inherited from parental PB‐NK cells to PB expanded NK (eNK) cells, with limited acquisition of cytotoxicity. (A and B) Comparison of the production IFN‐γ and TNF‐α, as well as CD107a expression, by PB‐NK and PB eNK cells in response to stimulation with 1× PMA and ionomycin (P/I) (A) or 2 µg/mL immobilized 3G8 (B) for 6 h. P/I stimulation groups: IFN‐γ: PB‐NK, *n* = 11; PB eNK, *n* = 11. TNF‐α: PB‐NK, *n* = 7; PB eNK, *n* = 7. CD107a: PB‐NK, *n* = 7; PB eNK, *n* = 7. 3G8 stimulation groups: IFN‐γ: PB‐NK, *n* = 8; PB eNK, *n* = 8. TNF‐α: PB‐NK, *n* = 8; PB eNK, *n* = 8. CD107a: PB‐NK, *n* = 8; PB eNK, *n* = 8. (C) Comparison of cytokine expression profile between PB ecNK and PB eg‐NK cells upon stimulation with 2 µg/mL immobilized 3G8 for 6 h (*n* = 10). (D and E) The MTT assay was employed to assess the proportion of viable target cells. HCT116 and MDA‐MB‐468 were used as target cells in coculturing with PB eNK cells at the depicted effector‐to‐target (E:T) ratios, with or without 10 µg/mL cetuximab (CET), to trigger ADCC‐mediated killing and natural cytotoxicity. (D) HCT116 alone, *n* = 2; the addition of CET, *n* = 4; E:T = 3:1, *n* = 4; E:T = 1:1, *n* = 4; E:T = 3:1 with CET, *n* = 4; E:T = 1:1 with CET, *n* = 4. (E) MDA‐MB‐468 alone, *n* = 3; the addition of CET, *n* = 3; E:T = 3:1, *n* = 6; E:T = 1:1, *n* = 6; E:T = 3:1 with CET, *n* = 6; E:T = 1:1 with CET, *n* = 6. (F) The distinct responses of PB ecNK and PB eg‐NK cells to MDA‐MB‐468 target cell stimulation, in addition to their reaction to CD16 pathway activation, were examined in the presence or absence of 2 µg/mL CET precoating. For the IFN‐γ, TNF‐α, and CD107a detection groups, each group contained five samples. (G) After incubation with PB eNK cells for 6 h (E:T = 5:1), with or without 10 µg/mL RTX, the survival of Daudi cells was evaluated by FCM‐based killing assay. PB eNK cells were categorized into g‐NK^high^ and g‐NK^low^ groups based on the percentage of FcRγ^–^ subset from twelve eNK cell batches (57.7 ± 6.67 versus 23.1 ± 9.20%). g‐NK^high^ group: *n* = 3; g‐NK^low^ group: *n* = 3. Dots linked by a line represent data collected from the same donor. Error bars indicate standard deviation (SD). The statistical significances were determined using two‐tailed unpaired and paired *t*‐tests, where *p < 0.05; **p < 0.01; ***p < 0.001; ****p < 0.0001.

Next, we explored the functional attributes of PB eNK cells. In resting state, only a few PB eNK cells expressed IFN‐γ, TNF‐α, and CD107a (Figure S7A). Upon stimulation with PMA and ionomycin (P/I) or 3G8 (an agonist mAb activating the CD16 pathway), PB eNK cells produced comparable levels of IFN‐γ and TNF‐α compared with primary PB‐NK cells (Figure 4A,B). However, significant differences were noted in CD107a expression, with PB eNK cells exhibiting higher degranulation capability following P/I stimulation but lower after 3G8 stimulation when compared with primary PB‐NK cells (Figure 4A,B). When encountering MDA‐MB‐468 cells, PB eNK cells produced IFN‐γ at level equivalent to primary PB‐NK cells but displayed elevated TNF‐α production (Figure S7B). While PB‐NK cells showed a marked increase in cytokines production after encountering cetuximab (CET)‐opsonized target cells, this was not the case for PB eNK cells, indicating a potential impairment in the CD16 intracellular signaling pathway that leads to weak cytokines production in these cells (Figure S7B). However, PB eNK cells possessed the capacity to kill target cells via ADCC manner (Figure 4D,E).

In resting condition, PB eg‐NK cells exhibited lower levels of expression for IFN‐γ, TNF‐α, and CD107a compared with PB ecNK cells (Figure S7C). Upon stimulation with 3G8, PB eg‐NK cells exhibited a significant elevation in the production of IFN‐γ and TNF‐α, while simultaneously exhibiting a decrease in CD107a expression, in comparison with PB ecNK cells (Figure 4C). The skewed cytokines production trends aligned with those observed in primary PB cNK and PB g‐NK cells.^27^

To further assess their cytotoxicity, we exposed PB eNK cells to two cell lines expressing EGFR, HCT116 and MDA‐MB‐468 (Figure 4D,E). PB eNK showed higher natural cytotoxicity against HCT116 cells compared with MDA‐MB‐468 cells. While CET minimally augmented ADCC‐mediated elimination of HCT116 cells, it was more effective against MDA‐MB‐468 cells (Figure 4D,E). To understand whether the ability to produce cytokines is maintained while exerting cytotoxicity, we analyzed the production of IFN‐γ and TNF‐α, as well as CD107a expression, in PB eNK cells in response to MDA‐MB‐468 cells with and without CET (Figure 4F). PB eg‐NK cells displayed comparable levels of cytokines production and reduced degranulation when exposed to MDA‐MB‐468 cells compared with PB ecNK cells. Interestingly, the addition of CET increased cytokines production in PB eg‐NK cells, albeit without enhancing degranulation. This phenomenon, however, was not observed in PB ecNK cells (Figure 4F). Furthermore, we confirmed the effective engagement of the CD16 pathway in PB ecNK cells in the presence of CET, evidenced by the downregulation of CD16 expression (Figure S7E). In summary, PB eNK cells maintained the capacity for IFN‐γ and TNF‐α production similar to their parental cells upon CD16 pathway activation, with PB eg‐NK cells demonstrating a stronger response than PB ecNK cells. However, the choice of stimulation methods had a crucial impact on their functional outcome. Additionally, PB eNK cells retained natural cytotoxicity and ADCC activity, although weaker than UCB eNK cells (see Section 2.5). This could be related to low expression of molecules on PB eNK cells involved in their cytotoxicity.

In comparison with primary PB‐NK cells, PB eNK cells displayed a decreased level of perforin while demonstrating an increased expression of Gzm B (Figure S7F,G). On the other hand, PB eg‐NK cells consistently exhibited lower expression of both perforin and Gzm B compared with PB ecNK cells (Figure S7H,I). Similarly, PB eg‐NK cells had consistently lower levels of FASL and TRAIL compared with PB ecNK cells (Figure S7J,K). These findings indicate that PB eg‐NK cells may possess lower cytotoxic capability than PB ecNK cells. To evaluate the killing potential of PB eg‐NK and PB ecNK cells, CFSE‐labeled PB eNK cells were incubated with unlabeled CD20^+^ Daudi cells, both in the presence and absence of the addition of anti‐CD20 rituximab (RTX). We selected six expansions and categorized them into groups of higher and lower PB eg‐NK cell proportions (Figure 4G). The results revealed that the proportion of g‐NK did not affect natural cytotoxicity. However, compared with batches with a lower proportion of g‐NK cells, PB eNK cell batches exhibiting a higher proportion of the g‐NK subset displayed a diminished capacity for ADCC‐mediated killing (Figure 4G). This observation could be attributed to the lower CD16 expression and cytotoxic molecular patterns exhibited by PB eg‐NK cells (Figures 3B and S7H–K).

### 2.4 Bulk NK and g‐NK subsets are expanded in primary UCB‐NK cells with PLH feeder cells

**FIGURE 5:**
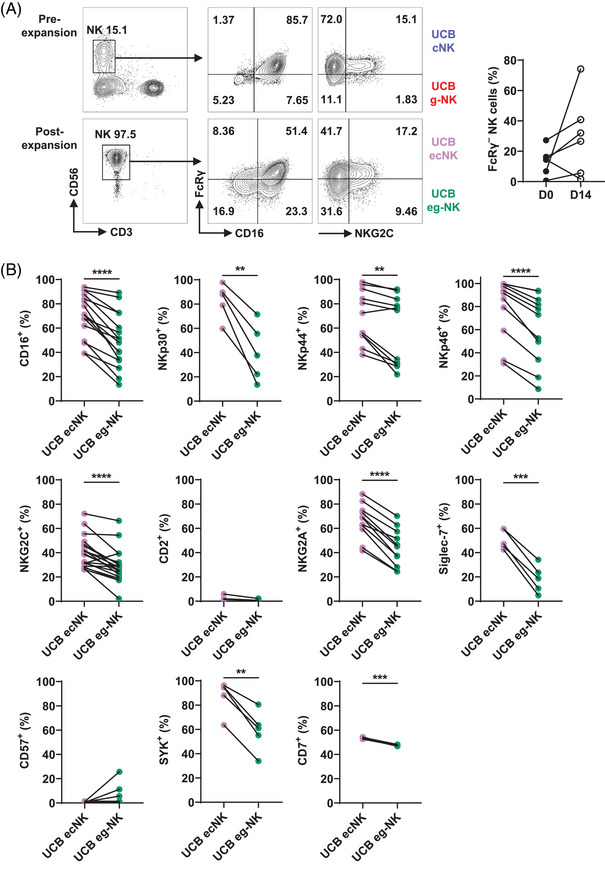
Expansion of bulk NK and g‐NK subsets in UCB‐NK cells with PLH feeder cells. (A) Representative plots showcasing UCB‐NK cells before and after 14 days of expansion. The left graph depicting FCM contour plots of UCB‐NK cells from a representative donor, both pre‐ and postexpansion. The right graph illustrating the proportional change in the UCB eg‐NK subset after 14 days of expansion (*n* = 6). (B) The expression of eleven phenotypic markers between UCB ecNK and UCB eg‐NK cells is shown here, including CD16 (*n* = 16), NKp30 (*n* = 5), NKp44 (*n* = 11), NKp46 (*n* = 11), NKG2C (*n* = 20), CD2 (*n* = 4), NKG2A (*n* = 11), Siglec‐7 (*n* = 5), CD57 (*n* = 11), SYK (*n* = 5), and CD7 (*n* = 4). Dots linked by a line represent data collected from the same donor. Two‐tailed paired *t*‐tests were used for all comparisons. **p < 0.01; ***p < 0.001; ****p < 0.0001.

The above results showed that PLH cells could preferentially stimulate and promote the proliferation of UCB‐NK cells, especially the NKG2C^+^ subset (Figures 1B and S5C). Crucially, PLH cell stimulation aided in maintaining the expression of activating receptors and the FcRγ^–^ phenotype in UCB‐NK cells (Figure 2B,D). Therefore, we selected PLH as feeder cells for the expansion of UCB‐NK cells and evaluated the phenotypic differences between UCB ecNK and UCB eg‐NK cells. Following a 14‐day stimulation, UCB eNK cells expanded up to 700‐fold^45^ (data not shown). CD16^+^ cells made up an average of 72.2 ± 14.8% of UCB eNK cells, which remained comparable to the 70.9 ± 19.3% observed in CD16^+^ primary UCB‐NK cells (Figure S8A). After expansion, the proportion of the UCB eg‐NK subset increased from 13.3 ± 9.05 to 30.1 ± 26.4% (Figure 5A). The mean proportion of eg‐NK cells among total UCB eNK cells was 32.7 ± 18.4%, with ecNK cells accounting for the rest (Figure S8C). The UCB eg‐NK subset could achieve a remarkable 287‐fold expansion in the PLH expansion system, far surpassing the initial number of primary UCB g‐NK cells (Figure S8D).

In primary UCB‐NK cells, UCB g‐NK cells express lower levels of CD16, NKG2A, SYK, NKp44, NKp46, CD2, and CD7, with no statistical differences in the expression of NKp30, NKG2C, and Siglec‐7 compared with UCB cNK cells.^27^ Analysis of these adaptive NK cell markers after expansion revealed that UCB eg‐NK cells exhibited lower expression of CD16, NKp30, NKp44, NKp46, NKG2C, NKG2A, Siglec‐7, SYK, and CD7 compared with UCB ecNK cells. However, there was no significant difference in the expression of CD2 (Figure 5B). Of note, primary UCB‐NK cells do not express CD57.^27^ Although a trend towards increased CD57 expression was observed in some UCB eg‐NK cells, this did not reach statistical significance (Figure 5B). These results indicate that UCB eNK cells exhibit a phenotype similar, although not identical, to primary UCB‐NK cells.

### 2.5 UCB eNK cells show a significant reduction in cytokines production capacity but acquire potent cytotoxicity after expansion

**FIGURE 6:**
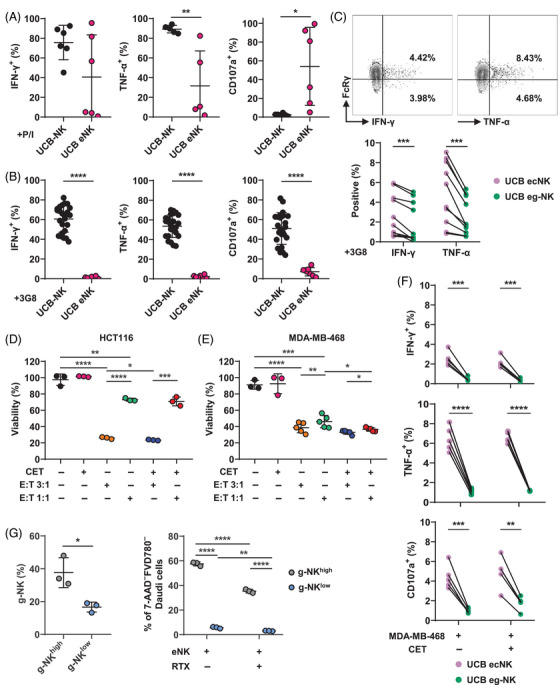
The reduced cytokines production contrasts with the potent cytotoxic potential observed in UCB eNK cells following expansion. (A and B) The production of IFN‐γ and TNF‐α, as well as CD107a expression, was assessed in UCB‐NK and UCB eNK cells upon stimulation with 1× (P/I) (A) or 2 µg/mL immobilized 3G8 (B) for 6 h. P/I stimulation groups: IFN‐γ: UCB‐NK, *n* = 6; UCB eNK, *n* = 6. TNF‐α: UCB‐NK, *n* = 5; UCB eNK, *n* = 5. CD107a: UCB‐NK, *n* = 6; UCB eNK, *n* = 6. 3G8 stimulation groups: IFN‐γ: UCB‐NK, *n* = 24; UCB eNK, *n* = 7. TNF‐α: UCB‐NK, *n* = 24; UCB eNK, *n* = 7. CD107a: UCB‐NK, *n* = 24; UCB eNK, *n* = 7. (C) Cytokine expression profile of UCB ecNK and UCB eg‐NK cells after stimulation with 2 µg/mL immobilized 3G8 for 6 h (*n* = 10). (D and E) UCB eNK cells and target cells, that is, HCT116 and MDA‐MB‐468, were cocultured at E:T ratios of 1:1 and 3:1. The ADCC and natural cytotoxicity of UCB eNK cells were assessed by precoating CET at 10 µg/mL for 1 h and omitting it, respectively. The target cell survival rate was analyzed through MTT assay. (D) HCT116 alone, *n* = 3; the addition of CET, *n* = 3; E:T = 3:1, *n* = 3; E:T = 1:1, *n* = 3; E:T = 3:1 with CET, *n* = 3; E:T = 1:1 with CET, *n* = 3. (E) MDA‐MB‐468 alone, *n* = 3; the addition of CET, *n* = 3; E:T = 3:1, *n* = 5; E:T = 1:1, *n* = 5; E:T = 3:1 with CET, *n* = 5; E:T = 1:1 with CET, *n* = 5. (F) UCB ecNK and UCB eg‐NK cells were cocultured with MDA‐MB‐468 cells, either in the absence or presence of 2 µg/mL CET. The production of IFN‐γ and TNF‐α, as well as CD107a expression, was measured by FCM after 6 h of stimulation (*n* = 5). (G) After incubation with UCB eNK cells for 6 h (E:T = 5:1), with or without 10 µg/mL RTX, the survival of Daudi cells was evaluated by FCM‐based killing assay. UCB eNK cells were categorized into g‐NK^high^ and g‐NK^low^ groups based on the percentage of FcRγ^–^ subset from six eNK cell batches (37.6 ± 9.11 versus 16.6 ± 3.04%). g‐NK^high^ group: *n* = 3; g‐NK^low^ group: *n* = 3. Dots linked by a line represent data collected from the same donor. Error bars indicate SD. The statistical significances were determined using two‐tailed unpaired and paired *t*‐tests, where *p < 0.05; **p < 0.01; ***p < 0.001; ****p < 0.0001.

The functional characteristics of UCB eNK cells were further assessed. In resting state, a minimal expression of IFN‐γ, TNF‐α, and CD107a was observed in both UCB‐NK and UCB eNK cells (Figure S9A). Upon P/I stimulation, UCB eNK cells exhibited a significant reduction in TNF‐α production compared with primary UCB‐NK cells, with no difference observed in IFN‐γ production. However, UCB eNK cells exhibited an enhanced degranulation capacity when compared with their pre‐expansion state (Figure 6A). Surprisingly, after activation of the CD16 pathway through 3G8 stimulation, UCB eNK cells displayed a drastic decrease in cytokines production and degranulation compared with UCB‐NK cells (Figure 6B). Even in the presence of CET, which activates the CD16 pathway via ADCC, UCB eNK cells produced lower levels of cytokines than UCB‐NK cells (Figure S9B).

In nonstimulated condition, UCB eg‐NK cells showed lower levels of IFN‐γ, TNF‐α, and CD107a than UCB ecNK cells (Figure S9C). Upon 3G8 stimulation, UCB ecNK cells consistently demonstrated higher production of IFN‐γ and TNF‐α relative to UCB eg‐NK cells, as well as an increased expression of CD107a (Figures 6C and S9D).

Next, the killing capacity of UCB eNK cells was evaluated. Interestingly, UCB eNK cells showed potent cytotoxicity against HCT116 and MDA‐MB‐468 cells compared with PB eNK cells (Figures 4D,E and 6D,E). This implies that UCB eNK cells are more akin to killer cells than cytokine producers. On the other hand, UCB eg‐NK cells showed reduced cytokines production and degranulation in response to MDA‐MB‐468 target cells, regardless of the presence or absence of CET (Figure 6F).

To investigate whether the enhanced cytotoxicity capability of UCB eNK cells is linked to the expression of cytotoxic molecules within them, we further evaluated the expression of perforin, Gzm B, FASL, and TRAIL (Figure S9F–K). Our findings revealed that UCB eNK cells displayed reduced level of perforin and elevated level of Gzm B when compared with their pre‐expansion state (Figure S9F,G). UCB eNK cell batches comprising a high g‐NK subset proportion exhibited a diminished natural cytotoxicity and ADCC against Daudi cells in contrast to batches with a low g‐NK subset proportion (Figure 6G). The relatively lower expression of perforin and Gzm B in UCB eg‐NK cells compared with ecNK cells could partially explain this phenomenon (Figure S9H,I). This pattern was similar to the expression of cytotoxic molecules observed in PB ecNK and PB eg‐NK cells. Additionally, UCB eg‐NK cells exhibited an increased expression of FASL but a decreased expression of TRAIL compared with UCB ecNK cells (Figure S9J,K).

### 2.6 The formation of the terminal function of eNK cells benefits from the stimulation and training provided by feeder cells

**FIGURE 7:**
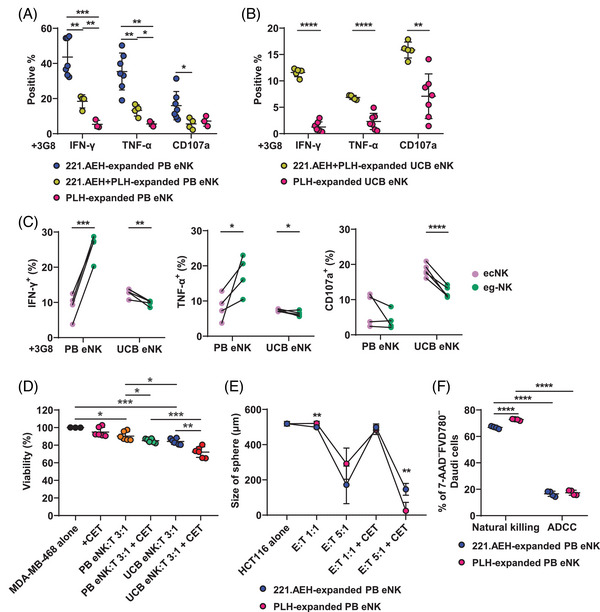
Feeder cell stimulation affects the development of terminal effector function in eNK cells. (A) PB eNK cells were expanded by 221.AEH, PLH, and a combination of both and stimulated with 2 µg/mL immobilized 3G8 for 6 h before analyzing cytokines production and CD107a expression by FCM. Scatter plot graphs showing the proportion of IFN‐γ^+^, TNF‐α^+^, and CD107a^+^ subsets within the PB eNK cells. Sample numbers: 221.AEH‐expanded PB eNK, *n* = 7; 221.AEH+PLH‐expanded PB eNK, *n* = 4; PLH‐expanded PB eNK, *n* = 3. (B) UCB eNK cells were analyzed as in graph (A) (221.AEH+PLH‐expanded UCB eNK, *n* = 5; PLH‐expanded UCB eNK, *n* = 7). (C) After 6 h of stimulation with 2 µg/mL immobilized 3G8, before–after plot graphs showing the proportion of IFN‐γ^+^, TNF‐α^+^, and CD107a^+^ subsets in ecNK and eg‐NK cells. These cells were obtained through expansion using a combination of feeder cells (221.AEH+PLH at a 1:1 ratio), encompassing both PB eNK (*n* = 4) and UCB eNK (*n* = 5) cells. (D) After coculturing with target cells for 24 h, the natural cytotoxicity and ADCC‐mediated killing of eNK cells were assessed in the presence or absence of 10 µg/mL CET. These eNK cells were expanded with combined feeder cells. The survival of MDA‐MB‐468 cells (corresponding to target cell (T) in graph) was analyzed by MTT assay. Sample numbers: MDA‐MB‐468 alone, *n* = 3; the addition of CET, *n* = 6; PB eNK:T = 3:1, *n* = 6; PB eNK:T = 3:1 with CET, *n* = 6; UCB eNK:T = 3:1, *n* = 6; UCB eNK:T = 3:1 with CET, *n* = 6. (E) PB eNK cells expanded with 221.AEH or PLH cells were incubated with HCT116 tumor spheres for 48 h at E:T ratios of 1:1 and 5:1 in presence of 10 µg/mL of CET or not. The extent of eNK‐mediated killing was assessed by comparing tumor sphere size. 221.AEH‐expanded PB eNK: HCT116 alone, *n* = 6; E:T = 1:1, *n* = 6; E:T = 5:1, *n* = 3; E:T = 1:1 with CET, *n* = 6; E:T = 5:1 with CET, *n* = 4. PLH‐expanded PB eNK: HCT116 alone, *n* = 6; E:T = 1:1, *n* = 5; E:T = 5:1, *n* = 6; E:T = 1:1 with CET, *n* = 6; E:T = 5:1 with CET, *n* = 4. (F) CFSE‐labeled PB eNK cells were coincubated with Daudi cells at an E:T ratio of 5:1. These eNK cells were expanded using 221.AEH or PLH feeder cells. After 6 h of incubation, FCM was used to measure the proportion of viable CFSE^–^ cells (7‐AAD^–^FVD780^–^) (221.AEH‐expanded PB eNK: *n* = 4; PLH‐expanded PB eNK: *n* = 4). Dots linked by a line represent data collected from the same donor. Error bars indicate SD. The statistical significances were determined using two‐tailed unpaired and paired *t*‐tests, where *p < 0.05; **p < 0.01; ***p < 0.001; ****p < 0.0001.

To investigate whether the production of cytokines or the formation of high cytotoxicity in eNK cells after expansion is a result of feeder cell stimulation, we attempted to adopt methods of switching feeder cells or using mixed feeder cell expansion to endow eNK cells with their missing functionality. Therefore, we tried to expand PB‐NK and UCB‐NK cells using PLH and 221.AEH cells, respectively. Although PLH cells effectively induced PB‐NK cell expansion, we noted a suboptimal viability ranging from 34% to 86% in PB eNK cells after a 15‐day expansion period. Additionally, the percentage of CD56^+^CD3^–^ NK cells in the PLH‐expanded PB eNK cell batches underwent notable fluctuations throughout the expansion process (Figure S10B). Similarly, 221.AEH cells exhibited suboptimal performance in the expansion of UCB‐NK cells, for example, were inferior to those achieved using the PLH expansion system. Moreover, eNK cells expanded with 221.AEH cells have difficulty surviving for more than 7 days, indicating a problem in the initiation of the expansion process (Figure S10C,D). These observations indicate that PLH cells may possess specific stimulatory properties for UCB‐NK cells, whereas 221.AEH cells may be a more suitable candidate for promoting the expansion of PB‐NK cells. Based on the above, we explored whether a combination of feeder cells (PLH and 221.AEH in a 1:1 ratio) could mitigate the limitations of the single feeder cell expansion system. After activating CD16 pathway with 3G8 stimulation, PB eNK cells expanded with combined feeder cells exhibited reduced production of IFN‐γ and TNF‐α compared with PB eNK cells expanded exclusively with 221.AEH cells (Figure 7A). Notably, the cytokines production capacity of PB eNK cells was nearly abolished when solely expanded with PLH feeder cells. UCB eNK cells expanded with combined feeder cells displayed elevated levels of IFN‐γ and TNF‐α production, as well as increased degranulation, in comparison with the counterpart expanded with PLH cells alone (Figure 7B).

After expanding PB‐NK and UCB‐NK cells with a combination of feeder cells, we further compared cytokines production upon 3G8 stimulation of ecNK and eg‐NK cells (Figure 7C). In PB eNK cells, the eg‐NK subset exhibited higher production of IFN‐γ and TNF‐α compared with the ecNK subset, while CD107a expression remained comparable. These results aligned with the cytokines production patterns observed in PB eNK cells expanded with 221.AEH cells (Figures 7C and 4C). Conversely, the UCB eg‐NK subset exhibited lower IFN‐γ and TNF‐α production and CD107a expression compared with the ecNK subset, resembling UCB eNK cells expanded with PLH alone (Figures 7C and 6C). Additionally, eNK cells expanded using combined feeder cells presented lower natural cytotoxicity and ADCC capacities against MDA‐MB‐468 cells compared with eNK cells expanded by the appropriate feeder cells, that is, A221.AEH and PLH for PB eNK and UCB eNK cells, respectively (Figures 4, 6, and 7D); though that, UCB eNK cells still maintained relatively higher cytotoxicity than PB eNK cells (Figure 7D). In short, although the 221.AEH expansion system contributed to higher cytokines production in UCB eNK cells, efforts to improve cytokines production in these cells were associated with a decrease in their cytotoxicity.

Figure 7D demonstrated that the use of feeder cell combination did not enhance the cytotoxic potential of PB eNK cells. To further explore this, we conducted additional experiments, evaluating the influence of PLH feeder cells on the killing activity of PB eNK cells towards HCT116 tumor spheres and Daudi cells (Figure 7E,F). In comparison with PB eNK cells that were expanded using 221.AEH cells, we observed a slight superior ADCC‐mediated killing activity among PB eNK cells expanded with PLH cells within the tumor sphere model (Figures 7E and S11A). However, these PLH‐expanded PB eNK cells exhibited a decreased natural cytotoxicity against Daudi cells (Figure 7F). Consequently, we found that the same protocol that effectively enhance the cytotoxicity of UCB eNK did not yield a significant increase in the cytotoxicity of PB eNK. This underscores that the feeder cells’ capacity to modulate NK cell function is contingent upon not just the specific expansion protocols but also the source of NK cells.

## 3 DISCUSSION

The fate of NK expansion is determined by several interconnected factors, encompassing the source of NK cells, the cytokine environment, the presence and type of feeder cells, along with the condition of samples (fresh or cryopreserved).^34^, ^47^, ^48^, ^49^ Cytokine‐induced NK cell expansion is very limited compared with feeder cell system,^34^ even using multiple cytokine combinations.^50^ The latter study demonstrates that the expansion efficiency with cytokines‐only stimulation is inversely proportional to the cytotoxic activity of the produced NK cells.^50^ This is the reason that now days more teams are focusing on using feeder cells, which enable significant expansion while maintaining high cytotoxic activity. Previous research has highlighted the role of feeder cells, such as K562 and 721.221, in modulating the functional properties of NK cells.^51^ K562 cells promote NK cell expansion by disrupting the inhibitory‐activating receptor balance, primarily due to their lack of ligands for inhibitory killer‐cell immunoglobulin‐like receptors expressed by NK cells. These feeder cells can endow NK cells with the ability to degranulate and lyse target cells. On the contrary, 721.221 cells interact with activating receptors, such as CD48, CD80, and CD86, expressed on the membrane of NK cells, furnishing costimulatory signals that promote the proliferation of NK cells into a subpopulation with improved cytokine‐secreting capability.^52^ Another study has shown that the CTV‐1 cells can preactivate resting NK cells and augment the cytolytic activity of activated NK cells.^30^ Compared with K562 cells, coculture with CTV‐1 cells results in NK cells producing higher levels of cytokines, including IFN‐γ, TNF‐α, MIP‐1α, RANTES, IL‐2Rα, and IL‐1β.^30^ After preactivation mediated by tumor cells, NK cells experience transcriptional alterations. For instance, K562‐preactivated NK cells exhibit downregulation of genes, such as *GZMA/B*, *HAVCR1/2*, *JAK1/3*, and *MAP3K2/3/5/9/10/12/13*. Conversely, exposure to CTV‐1 or IL‐2 induces a gene expression profile partially overlapping with those linked to enhanced effector function, including upregulation of *FAS*, *TNSF10*, *CD69*, *CD80*, *CD83*, and *MAPK11*, as well as cytokine‐encoding genes like *TNF*, *IFNG*, *CCL3/4/8*, and *CXCL9/11*.^30^ These findings underscore the critical impact of specific feeder cells in shaping the functional attributes of NK cells, ultimately affecting their cytotoxic and cytokine‐secreting potentials.

K562 cells have been described as capable of increasing the percentage of NKG2C^+^ NK subset and expanding this subset, particularly when overexpressing HLA‐E.^43^ In fact, K562 cells express a certain amount of HLA‐E.^53^ PLH and 721.221 cells are EBV‐transformed B‐lymphoblastoid cell lines, whereas K562 cells are derived from chronic myelogenous leukemia blast.^52^, ^54^, ^55^ Interestingly, NKG2C^hi^CD57^+^ NK cells respond specifically to acute infection with HCMV and not EBV, and furthermore, the NKG2C^hi^ NK subset does not experience expansion following EBV infection.^56^ Therefore, our results in vitro agree to those described earlier in vivo, in contrast to K562 cells, PLH cells do not induce NKG2C upregulation. Additionally, K562 cell stimulation could enrich the FcRγ^–^ subset in PB‐NK cells, but not in UCB‐NK cells. Despite our incomplete comprehension of the exact mechanism, as earlier stated, K562 cells could prompt an increase in the percentage of NKG2C^+^ NK cells, which have been associated with the FcRγ^–^ NK subset. Consequently, the observed enrichment in the FcRγ^–^ NK subset in PB‐NK cells, and not in UCB‐NK cells, could be attributed to the elevated NKG2C^+^ NK subset in PB‐NK cells. This phenomenon could stem from the fact that PB‐NK cells are more mature than UCB‐NK cells, thereby exhibiting a more robust response to the interaction with K562 cells. The precise molecular expression patterns and stimulatory signals mediated by PLH as feeder cells remain elusive, although PLH cells have emerged as a favorable approach for expanding UCB‐NK cells.^44^, ^45^ Previous studies have highlighted the capacity of HLA‐E‐overexpressing feeder cells to facilitate the expansion and activation of NKG2C^+^ and FcRγ^–^ NK cells.^41^, ^42^, ^43^, ^57^ Our results revealed that 221.AEH cells enhanced the proportion of both NKG2C^+^ and NKG2C^–^ NK subsets, both belonging to the FcRγ^–^ subset. We uncovered that PLH cells exceled at expanding UCB‐NK cells, whereas 221.AEH cells were a more suitable choice for the expansion of PB‐NK cells. Subsequently, we elucidated the influence of these feeder cells on NK cell phenotype. Ultimately, through the implementation of a synergistic feeder cell strategy to expand PB‐NK and UCB‐NK cells, we observed that the phenotype and functionality imparted by the primary feeder cells, such as PLH cells for UCB‐NK and 221.AEH cells for PB‐NK, is firmly established and barely altered by the second option of feeder cells.

Exposing NK cells to target cells can downregulate the expression of multiple activation receptors, including NKG2D, CD16, NKp30, and NKp46.^30^ We observed that after stimulation by K562 and 221.AEH cells, PB‐NK and UCB‐NK cells downregulated natural cytotoxicity receptors (NCRs), including NKp30, NKp44, and NKp46, and NKG2D. When compared with unstimulated, that is, D0, the NF group and the PLH cell stimulation group demonstrated a significant increase in the expression of NCRs and NKG2D. A possible explanation is the presence of activating receptors’ ligands on feeder cells capable of releasing soluble ligands likely accounting for the temporary downregulation of these NK cell receptors. Downregulation of NKG2D is due to its binding with membrane‐bound or soluble ligands, which induces clathrin‐mediated endocytosis and activates the ERK1/2 signaling pathway through DAP10.^58^, ^59^ In addition, upon conjugation with MICA‐expressing target cells, MICA can be transferred to NK cells through cell contact, accumulating at the immunological synapse. This transfer is facilitated by NKG2D‐specific molecular recognition and augmented by Src kinase signaling. The orientation of MICA binding to its new host NK cell membrane allows for trans‐binding with NKG2D, resulting in the downregulation of NKG2D in subsequent homotypic interactions with other NK cells.^60^ However, wild‐type (WT) 721.221 cells do not express any NKG2D ligands, so the mechanisms underlying NKG2D downregulation on NK cells induced by them remain unknown. Sabry et al.^30^ found that PB‐NK cells stimulated with K562 cells overnight always express lower levels of NCRs than PB‐NK cells stimulated with IL‐2/12/15/18 (individually or in combination). This is consistent with our data. However, we only have the time‐point of day 3 (D3) and there is no dynamic expression change of NCRs and NKG2D under different stimulation conditions. The intracellular regulatory mechanisms, such as tyrosine phosphorylated molecules, that mediate its changes are unclear. In addition, we observed that after 14 days of expansion, PLH‐expanded UCB eNK cells lacked proper expression of NKG2D and NKp46, but highly expressed NKp44 (data not shown). So, the phenotype of NK cells is constantly changing from short‐term activation to long‐term expansion. The downregulation of a specific activating receptor does not fully determine eNK cell function, further complicating the functional regulatory mechanisms.

The simultaneous presence of cytokines production and cytotoxicity, especially after in vitro NK cell expansion, remains unclear. During the expansion process, microenvironmental stimulations play a pivotal role in shaping NK cell fate, dictating whether they become anergic or develop into potent cytotoxic effectors. Previous investigations have provided insights into the compartmentalization of cytokines and cytotoxic molecules in different cellular compartments. Cytokines are transported and released by the recycling endosome, while perforin and Gzm B are mainly stored in cytotoxic secretory vesicles. The release of perforin occurs in a polarized manner at the cell synapse, whereas cytokines like IFN‐γ and TNF‐α are transported to various locations on the cell surface for nonpolarized release, including within the synapse.^8^ Cooper et al.^61^ reported that CD56^bright^CD16^–^ NK cells produce abundant cytokines but mainly lack cytotoxic activity; in contrast, CD56^dim^CD16^+^ NK cells show robust cytotoxic activity while producing significantly lower levels of cytokines. This is the best‐known example that cytokines production and cytolytic capacity are not linked in NK cells, but there are others. An early study in IFN‐γR knockout mice demonstrate that IFN‐γ is not involved in the killer activity of IL‐12/IL‐18‐derived NK cells, which kill through perforin‐mediated mechanism.^62^ In addition, Anft et al.^63^ found that NK cells that fail to efficiently kill target cells can still significantly influence immune response by enhancing the secretion of proinflammatory cytokines. Delayed detachment due to reduced NK cell cytotoxicity increases sustained calcium signaling in NK cells, which may be the cause of enhanced cytokine secretion.^63^ In primary PB‐NK cells, we noticed a dissociation between degranulation and cytokines production. Conversely, UCB‐NK cells tended to produce cytokines and degranulate concurrently. However, following expansion, eNK cells from both sources primarily specialized in either cytokines production or cytotoxic function. Therefore, our findings align with the previous notion that eNK cells incline to specialize in one function during chronic stimulation and expansion. PB eNK cells retained the properties of primary PB‐NK cells, demonstrating varied cytokines production patterns between PB ecNK and PB eg‐NK cells upon stimulation with immobilized 3G8. However, after expansion, PB eNK cells displayed a constrained capacity to produce IFN‐γ and TNF‐α in response to the stimulation of target cells opsonized with WT mAb (CET) (Figure 4F), indicating a need for refinement in the stimulation approaches. For instance, utilizing the engineered Fc variant mAbs (S239**D**/H268**F**/S324**T**/I332**E**),^64^ our preliminary data demonstrated that these mAbs could maximally stimulate the cytokine‐producing potential of PB eg‐NK cells. This strategy addresses the issue of PB eg‐NK cells’ low responsiveness in the CD16 pathway, which is of significant importance for applications in in vivo models. In contrast, UCB eNK cells displayed minimal cytokines production regardless of the stimulation methods. The downregulation of perforin expression suggests that postexpansion eNK cells may rely on alternative cytotoxic mechanisms, beyond membrane perforation, to eliminate target cells. In summary, cytokines production and cytolytic activity are shared by eNK cells, yet cells with high cytolytic activity tend to produce a lower level of cytokines, and vice versa, cells that produce high levels of cytokines tend to exhibit low cytotoxicity.

Our study sheds light on the phenotypic expression and functionality of cNK and g‐NK cells, derived from PB and UCB, following their expansion process. Notably, we highlight the influence of their origin and stimulatory condition on their functional properties. In view of the pivotal role in vitro NK cell expansion plays in innovative clinical applications, our research holds the potential to assist researchers and clinicians in devising alternative NK cell expansion strategies tailored to diverse clinical settings. NK cells that demonstrate robust cytokines production are crucial in orchestrating anti‐tumor immune responses, particularly in immune‐suppressive microenvironments.^65^, ^66^ Conversely, NK cells with potent cytotoxicity act as efficient tumor cell eliminator, while minimizing the risk of cytokine storms due to their limited cytokines production, rendering them an appealing choice for certain anti‐tumor therapeutic approaches. Consequently, a thorough comprehension of NK cells’ origin, expansion, function, and intricacy is essential for clinical application. However, our study has several limitations:

1. The molecular signals and mechanisms that underlie the functional formation of eNK cells due to the training of different feeder cells remain unknown. Remarkably, the mechanisms that result in diverse function following in vitro expansion are not attributable to a single factor. The complex balance of activating and inhibitory receptors that triggers NK cell activation poses significant challenges in identification, which surpasses the scope of our current work. Moreover, the utilization of primary samples introduces an additional layer of complexity, as donors vary in their NK cell subsets and the expression of activating and inhibitory receptors. Hence, the same feeder cell could use different mechanisms to stimulate NK cells from different donors, significantly complicating the identification of a universal mechanism.
2. Our analysis of g‐NK cells is hampered by the absence of specific extracellular g‐NK cell markers, thereby restricting our evaluation to the phenotype and function of eg‐NK cells within the broader eNK cell population postexpansion, particularly with regard to the cytotoxicity of purified eg‐NK cells.
3. A more comprehensive analysis is required to understand the influence of various blood sources and expansion systems on the in vivo persistence and tumor‐killing capability of eNK cells. Although we have extensively published the in vivo activity of PLH‐expanded UCB eNK cells,^44^, ^67^, ^68^, ^69^ the specific function of PB eNK cells in vivo remain to be elucidated. By mutating the amino acid residues at the complement‐binding site of the mAbs segment, we have developed mutated mAbs that can mediate enhanced ADCC and tightly bind to CD16.^64^ These mAbs coupled with NK‐001 (UCB eNK cells) are used for a clinical trial in lymphoma patients experiencing relapse following CAR‐T cell therapy (https://www.emercell.com/). Furthermore, CYT‐102 (Fc variant of anti‐EGFR mAbs armed UCB eNK cells) will be applied in the treatment of glioblastoma multiforme and other EGFR^+^ solid tumors (https://cytea.bio/). On the other hand, Bigley et al.^17^ observed that PB eg‐NK cells exhibit greater persistence in the blood and spleen compared with PB ecNK cells in vivo, and the combination of daratumumab with PB eg‐NK cells result in a 99.9% tumor reduction in a disseminated orthotopic xenograft MM.1S model of multiple myeloma, outperforming the combination with PB ecNK cells. However, during the in vitro expansion of g‐NK and cNK cells within PB, they are cultured with a lymphoblastoid cell line (specific name undisclosed) and K562‐mbIL15‐41BBL feeder cells, respectively. This potentially impart distinct functional traits to these cell types prior to adoptive therapy.^17^ Due to the fundamental differences in functional properties between PB eNK and UCB eNK cells, we are unable to determine which is superior in an in vivo model. A tailored tumor model must be designed, along with the selection of suitable mAbs and infusion of primary NK cell‐depleted PBMCs. The upcoming assessment will encompass mouse survival, tumor elimination, eNK cell infiltration in the tumor site, as well as the recruitment, percentage, phenotype, and function of various immune cell types within the TME, including macrophages, myeloid‐derived suppressor cells, dendritic cells (DCs), and various T cell subtypes. The ultimate aim is going to confirm the in vivo regulatory role of PB eg‐NK cells, known for their proficiency in producing IFN‐γ and TNF‐α. This is our future effort direction.
4. How can we establish a standardized screening reference for adoptive therapy based on eNK cells in the future? Before proceeding with adoptive therapy, it is imperative to initially assess the proportion and functionality of cNK and g‐NK cells within the donor's NK cell population. By integrating data derived from in vivo mouse models, we aim to determine whether g‐NK cells effectively control tumor growth or exhibit a weaker tumoricidal capacity among PB‐NK and UCB‐NK cells. Furthermore, the disease progression of the intended patient cohort must be taken into account. We still have a long way to go in this regard.

In conclusion, the complexity of NK cell functions suggest that their in vitro expansion must be adapted to their future clinical use. We unveil here feeder cell‐based protocols to direct eNK cells to specific functions. PB‐NK cells can be programmed to become cytokine producers after their expansion with 221.AEH cells, while UCB‐NK cells become highly cytolytic after using PLH as feeder cells. We envision those other conditions could generate additional NK cell subsets, rendering NK cell expansion protocols a compelling area of investigation. Our results underscore the significance of selecting feeder cells for NK cell expansion from various sources, notably for customized adoptive cell therapies to yield cytokine‐producing or cytotoxic eNK cells.

## 4 MATERIALS AND METHODS

### 4.1 Human blood samples

PB or buffy coat samples were primarily obtained from the “Etablissement Français du Sang (EFS),” France. CBUs were sourced from the Biological Resource Center Collection of the University Hospital of Montpellier—(BIOBANQUES Identifier—BB‐0033‐00031, CHU Montpellier). Isolation of peripheral blood mononuclear cells (PBMCs) and umbilical cord blood mononuclear cells (UCBMCs) was accomplished through density gradient centrifugation utilizing Ficoll (Cytiva), followed by cryopreservation in fetal bovine serum (FBS) from Gibco, supplemented with 10% DMSO (Miltenyi). This cryopreservation process involved a gradual temperature reduction at a rate of 1°C/min down to −80°C, ultimately saving in liquid nitrogen.

### 4.2 Cell lines

The utilization and cultivation of all cell lines were elaborated in the Supporting Information, Section 1.1.

### 4.3 NK‐cell immunophenotyping and functional analysis by multiparametric flow cytometry

The phenotype and functional analyses of NK cells were detailed in Supporting Information, Section 1.2.

### 4.4 Assessing the response of NK cells to feeder cells

The current study refined a protocol based on methodologies described by Tremblay‐McLean et al.^53^ and Lisovsky et al.^52^ These research entailed coculturing PBMCs/UCBMCs or eNK cells with K562, PLH, or 221.AEH cells at an effector‐to‐target (E:T) ratio of 2:1 for 6 h. At the outset of the coculture, brefeldin A (golgiPlug; BD), monensin (golgiStop; BD), and VioBlue‐conjugated anti‐CD107a monoclonal antibody (mAb) were added. Cells were processed for extracellular staining using PE‐Vio770‐conjugated anti‐CD56 (Miltenyi) and APC‐conjugated anti‐CD3 (Miltenyi) mAbs and Fixable Viability Dye eFluor™ 780 (FVD780; ThermoFisher). Subsequent to fixation and permeabilization, intracellular staining was conducted using PE‐conjugated anti‐IFN‐γ (Biolegend), and PerCP‐Cy5.5‐conjugated anti‐TNF‐α (Biolegend) mAbs. Data acquisition was performed on Canto II flow cytometer using FacsDiva™ (BD Biosciences), and analysis was executed using FlowJo™ V10.8.1.

### 4.5 NK‐cell isolation and expansion

The EasySep™ Human CD3 Positive Isolation Kit II (STEMCELL) was employed to deplete CD3^+^ T and NKT cells, leaving residual cells intact, including NK cells, monocytes, and DCs, for subsequent expansion. The non‐NK cells were conserved as autologous precursors and accessory cells, while irradiated 221.AEH or PLH cells (at a dose of 75 Gy, X‐ray irradiator; Xstahl) provided stimulatory and activated signals to NK cells. For details, please refer to the Supporting Information, Section 1.3.

### 4.6 Activation of CD16 pathway

The method for activating the CD16 pathway primarily involved directly stimulating the CD16 receptor using anti‐CD16 agonist 3G8 (Biolegend). Alternatively, activation can be achieved by precoating MDA‐MB‐468 cells with CET (Merck) to expose the Fc segment of mAb for binding to the CD16 receptor. For details, please refer to the Supporting Information, Section 1.4.

### 4.7 Cytotoxicity assays

The method for detecting eNK cell cytotoxicity included the MTT assay, which was used to assess the viability of target cells (e.g., MDA‐MB‐468 and HCT116) after coincubating with eNK cells for 24 h. Furthermore, flow cytometry (FCM) analysis determined the percentage of surviving Daudi cells following coculturing with eNK cells for 6 h. To further evaluate the efficacy of eNK cells in killing HCT116 cells, a 3D tumor sphere model was implemented, both in the presence and absence of CET. For a detailed outline of the procedures, please refer to the Supporting Information, Section 1.5.

### 4.8 Statistical analysis

Data were analyzed using Excel and GraphPad Prism 8, employing Student's *t*‐tests (paired and unpaired) where applicable. Significance levels were denoted as follows: *p < 0.05; **p < 0.01; ***p < 0.001; ****p < 0.0001. After examining the data, it was considered reasonable to assume that they follow a normal distribution. While verifying normality may be challenging with a small sample size, numerous other studies have conducted extensive measurements of similar parameters and concluded that the parameters are indeed normally distributed. Consequently, the decision was made to employ parametric tests for the statistical analysis.

## AUTHOR CONTRIBUTIONS

*Conceptualization*: Martin Villalba and Fei Gao *Methodology*: Fei Gao *Data analysis and interpretation*: Fei Gao *Initial writing*: Fei Gao *Correction*: Mauricio Campos Mora and Martin Villalba *Samples collection*: Michael Constantinides, Loïs Coënon, Caroline Multrier, Loïc Vaillant, and Julien Peyroux *Reviewing and editing*: Martin Villalba and Tianxiang Zhang *Supervision*: Martin Villalba and Tianxiang Zhang. All authors have read and approved the final manuscript.

## CONFLICT OF INTEREST STATEMENT

The authors declare no conflict of interest.

## ETHICS STATEMENT

The blood samples used in this study were strictly intended for scientific investigation. Before initiating the experiments, we received ethical clearance from the French National Ethics Committee (Approval No. 21PLER2018‐0069 for PB and BB‐0033‐00031 for CBUs). Each donor provided informed consent by signing a consent form prior to blood donation.

## Supporting information



Supporting Information

## Data Availability

With the consent of the corresponding author, the data in this article are shared.
