# Supplementary material for: Feeder cell training shapes the phenotype and function of in vitro expanded natural killer cells
Source: MedComm (2020). 2024 Sep 23;5(10):e740. doi: 10.1002/mco2.740 (PMC11417427; doi:10.1002/mco2.740)
Supplement: Supplementary file 1 — Supporting Information [file MCO2-5-e740-s001.docx]

Supplemental Materials

Feeder cell training shapes the phenotype and function of *in vitro* expanded natural killer (NK) cells

Fei Gao^1,2^, Mauricio Campos Mora^1^, Michael Constantinides^1^, Loïs Coënon^1^, Caroline Multrier^1^, Loïc Vaillant^1^, Julien Peyroux^1^, Tianxiang Zhang^3,^*, Martin Villalba^1,4,5,^*

1. IRMB, University of Montpellier, INSERM, CHR Montpellier, Montpellier, France;2. Department of Pathology, School of Basic Medicine, Central South University, Changsha, China; 3. Department of Immunobiology, Yale University School of Medicine, New Haven, Connecticut, USA; 4. Institut du Cancer Avignon-Provence Sainte Catherine, Avignon, France; 5. IRMB, Univ Montpellier, INSERM, CHU Montpellier, CNRS, Montpellier, France.

*martin.villalba@inserm.fr; tianxiang.zhang@yale.edu

**1. Supplemental experimental procedures**

## 1.1 Cell lines

All cell lines were obtained from commercial suppliers or directly from researchers that generated them. Hematopoietic cell lines were grown in RPMI 1640 glutamax™ (Gibco) supplemented with 10% FBS, including Daudi (human Burkitt’s lymphoma cell line, ATCC), K562 (human immortalized myelogenous leukemia cell line, ATCC), K562.AEH (constructed by Aoao Chen, Central South University, Changsha, China), 221.AEH (graciously provided by Dr. Miguel López-Botet’s laboratory, Pompeu Fabra University, Hospital del Mar Medical Research Institute, Barcelona, Spain), and PLH (EBV transformed lymphoblastoid line, ECACC). 221.AEH cells stably express a hybrid HLA-E and are derived from the human HLA-negative B-lymphoblastoid cell line 721.221. The preparation principle of K562.AEH is the same as that of 221.AEH ^1^. Other cell lines were cultured in high-glucose DMEM medium (Gibco) supplemented with 10% FBS, including MDA-MB-468 (human breast adenocarcinoma cell line, ATCC) and HCT116 (human colon carcinoma cell line, ATCC). The above cell lines were cultured without antibiotics and routinely tested for mycoplasma contamination. Of note, we discarded the cells after 3 months in culture and thawed a new batch of cells. We continued to grow most of the cell lines used in this study and we did not observe any difference compared to the original cells.

## 1.2 NK-cell immunophenotyping and functional analysis by multiparametric FCM

*Immunophenotyping:* Dead cells were excluded using Fixable Viability Dye eFluor™ 780 (FVD780, ThermoFisher) and 7-Aminoactinomycin D (7-AAD, Miltenyi). Extracellular markers for NK cell phenotype as follows: V500-conjugated anti-CD16 (#561394, BD), V480-conjugated anti-CD16 (#566171, BD), VioBlue-conjugated anti-CD16 (#130-113-396, Miltenyi), APC-conjugated anti-CD56 (#130-113-310, Miltenyi; IM2474U, Beckman), PE-Vio770-conjugated anti-CD56 (#130-113-313, Miltenyi), BUV496-conjugated anti-CD56 (#750479, BD), PerCP-Vio700-conjugated anti-CD56 (#130-114-551, Miltenyi), APC-conjugated anti-CD3 (#130-113-135, Miltenyi), PE-conjugated anti-CD3 (#130-113-139, Miltenyi), PerCP-Vio700-conjugated anti-CD3 (#130-113-141, Miltenyi), VioBlue-conjugated anti-CD3 (#130-114-710, Miltenyi), APC-Cy7-conjugated anti-CD3 (#300426, Biolegend), PE-Vio 615-conjugated anti-CD3 (#300426, Miltenyi), PE-conjugated anti-NKp30 (#130-092-483, Miltenyi), BV421-conjugated anti-NKp30 (#563385, BD), PE-conjugated anti-NKp44 (#130-092-480, Miltenyi), PE-conjugated anti-NKp46 (#557991, BD), VioBright515-conjugated anti-NKp46 (#130-112-278, Miltenyi), PE-Vio770-conjugated anti-NKp46 (#130-104-201, Miltenyi), PE-Vio770-conjugated anti-Siglec-7 (#130-100-977, Miltenyi), APC-conjugated anti-NKG2C (#130-117-398, Miltenyi), PE-conjugated anti-NKG2A (#130-098-814, Miltenyi), BB700-conjugated anti-NKG2A (#747926, BD), Pacific Blue-conjugated anti-CD57 (#A74779, Beckman), APC-conjugated anti-CD2 (#21380026S, Immunotools), FITC-conjugated anti-CD2 (#A07743, Beckman), PE-conjugated anti-CD7 (#IM9429U, Immunotools), Pacific Blue-conjugated anti-CD7 (#130-106-459, Miltenyi), PE-conjugated anti-CD69 (#130-112-613, Miltenyi), APC-Vio770-conjugated anti-CD19 (#130-113-643, Miltenyi), VioGreen-conjugated anti-CD45 (#130-110-638, Miltenyi), BV711-conjugated anti-FASL (#744101, BD), and BV786-conjugated anti-TRAIL (#743723, BD). Normally, 1 μL of antibodies (Abs) was mixed into 200 μL of 1× PBS containing 2% FBS, and each sample required 50 μL of this Abs mixture.

Approximately 2×10^5^ cells were incubated with the designated Abs in V-bottom 96-well plates (Falcon) at 4°C for 20 minutes. After washing and resuspending cells, each well was treated with 50 μL of fixation buffer (#554714, BD) for fixation at 4°C in the dark for 30 minutes. This was followed by centrifugation, resuspension, and the addition of 100 μL of 1× permeabilization buffer (#554714, BD) overnight. Abs recognizing several intracellular markers were then added, which included APC-conjugated anti-SYK (#130-099-283, Miltenyi), FITC-conjugated anti-FcRγ (#FCABS400F, Milli-Mark), anti-FcRγ (#06-727, Milli-Mark) polyclonal Ab labeled with Alexa Fluor® 647 Conjugation Kit (#ab269823, Abcam), PE-CF594-conjugated anti-perforin (#563763, BD), and PE-conjugated anti-granzyme B (Gzm B) (#130-101-351, Miltenyi). Intracellular staining was conducted in the dark at room temperature for 20 minutes, followed by 2 times washing. Subsequently, cells were resuspended in 300-500 μL of 1× PBS and analyzed using a BD Symphony A3 or Canto II flow cytometer. Acquisition was carried out using FacsDiva™ (BD Biosciences). Data analysis was performed using FlowJo™ V10.8.1.

*NK-cell cytokines production and degranulation:* The CD16 pathway was activated either with immobilized 3G8 or by target cells opsonized with specific monoclonal Abs (mAbs). After stimulation for 6 hours, cells were centrifuged and washed once with 1× PBS containing 2% FBS. The positive control group experienced cocktail stimulation involving 1× phorbol 12-myristate 13-acetate (PMA) and Ionomycin (#550583, BD Pharmingen™), abbreviated as P/I. The extracellular staining panel for analysis included FVD780, V500-conjugated anti-CD16, APC-conjugated anti-CD3, and PE-Vio770-conjugated anti-CD56 mAbs. Following fixation and permeabilization, intracellular staining was performed using a panel comprising FITC-conjugated anti-FcRγ, PE-conjugated anti-IFN-γ (#502509, Biolegend), and PerCP-Cy5.5-conjugated anti-TNF-α (#502926, Biolegend) Abs. VioBlue-conjugated anti-CD107a mAb (#130-111-628, Miltenyi) was added at the initiation of stimulation. Data were acquired using FacsDiva™ (BD Biosciences) on Canto II flow cytometer and then analyzed using FlowJo™ V10.8.1.

## 1.3 NK-cell isolation and expansion

*Single feeder cell expansion system:* After the removal of CD3^+^ T and NKT cells from PBMCs or UCBMCs, residual cells were preserved for subsequent expansion, including monocytes and dendritic cells (DCs) in addition to lymphocytes. These additional populations have been shown to better support NK cell expansion and survival ^2-4^. Even if CD3^+^ selection eliminated more T and NKT cells, some of them remained and they can be expanded alongside to NK cells (see for example Figure 3A). Typically, PBMCs consist of lymphocytes (T cells, B cells, and NK cells), monocytes, and DCs, with the frequencies of these subsets varying among individuals. In humans, lymphocytes typically comprise 70-90%, monocytes 10-20%, and DCs are rare, accounting for only 1-2% ^5^. Post-sorting, the purity of NK cells generally ranges from 5% to 30%, with most samples falling between 10% and 20%. Following sorting, we routinely employed the Muse cell analyzer (Luminex Guava) to quantify the proportion and count of CD56^+^ NK cells, monitoring them in real-time from day 0 (D0) to D14. For the detection of cytokine production, we typically used FCM, focusing on CD56^+^CD3^–^ NK cells. Therefore, even if trace amounts of CD3^+^ T cells and NKT cells remain after expansion, they did not impact our detection results. This logic was also applied to phenotypic detection.

X-ray irradiated K562.AEH, 221.AEH, and PLH cells (at a dose of 75 Gy, X-ray irradiator, Xstahl) were served as the primary feeder cells and provided stimulatory and activating signals to NK cells. Specifically, K562.AEH and 221.AEH cells were predominantly employed for expanding PB-NK cells, whereas PLH cells were used for expanding UCB-NK cells.

The NK cell culture medium was prepared by supplementing RPMI 1640 glutamax™ (Gibco) with 100 IU/mL IL-2 (#200-02-500UG, PeproTech), 5 ng/mL IL-15 (#130-096-491, Miltenyi), 5% human serum (Sigma), and 10% FBS (Gibco). Feeder cells were introduced at a ratio of 1:4 (NK:PLH) or 5:1 (NK:221.AEH or NK:K562.AEH) on D0 of expansion. To minimize disruption of NK cell activation, the NK medium was partially exchanged on D3. On D7, half of the culture medium was discarded, and cells were resuspended and stained with PE-conjugated anti-CD56 mAb (#130-113-312, Miltenyi) and Muse Count & Viability buffer (Luminex). The Guava® Muse® Cell Analyzer (Luminex) was employed to assess cell viability, cell number, and NK cell proportion. During the expansion period, the NK medium was partially refreshed, and feeder cells were replenished at ratios of 1:2 (NK:PLH) every 2-3 days or 1:1 (NK:221.AEH or NK:K562.AEH) every 5 days. After 14 days, NK cells underwent enrichment and expansion. The specific culture process and feeder cell addition followed the method outlined in previous studies ^3,6^.

*Combined feeder cell expansion system:* After irradiation (at a dose of 75 Gy), a 1:1 mixture of 221.AEH and PLH feeder cells was used to expand PB-NK or UCB-NK cells. The expansion protocol was the same as single feeder cell expansion system, with the exception of feeder cell substitution ^3^.

## 1.4 Activation of CD16 pathway

*Immobilized 3G8 mAb-based Assay:* For the stimulation of primary NK or eNK cells, MaxiSorp™ ELISA Plates (#423501, Biolegend) were pre-coated with 2 μg/mL anti-CD16 agonist 3G8 (#302049, Biolegend). Subsequently, cells were cultured in these plates. Brefeldin A (BFA, golgiPlug, BD), monensin (golgiStop, BD), and VioBlue-conjugated anti-CD107a mAb were added to the respective wells at the beginning of stimulation. After 6 hours, cells were harvested for phenotypic and functional assessment shown as supplemental material 1.2.

*Classic ADCC:* MDA-MB-468 cells, characterized as EGFR^+^, were seeded in a 96-well flat-bottom plate (Falcon) at a density of 30,000 cells per well, 1 day prior to the experiment. The following day, the culture supernatant was replaced with or without 100 μL 2 μg/mL cetuximab (CET, Merck) per well. After 1-hour incubation at 37°C in a 5% CO_2_ incubator, the supernatant was aspirated, and unbound mAbs were gently washed away using 1× PBS. Subsequently, primary NK or eNK cells were introduced into the respective wells. Simultaneously, BFA, monensin, and VioBlue-conjugated anti-CD107a mAb were added. The subsequent steps were executed as outlined above.

## 1.5 Cytotoxicity assays

*MTT Assay for Cytotoxicity Evaluation:* EGFR^+^ target cells (e.g., MDA-MB-468 and HCT116) were seeded at a density of 50,000 cells per well in 48-well flat-bottom plates (Falcon) and pre-incubated with 10 μg/mL CET for 1 hour at 37°C in a 5% CO_2_ incubator. Then they were co-incubated with PB eNK or UCB eNK cells for 24 hours. NK cell cytotoxicity was assessed by measuring target cell viability using the MTT assay, according to the protocol outlined by Sanchez-Martinez et al ^3^. In the absence of CET, cytotoxicity represents natural killing, in its presence, ADCC. For cytotoxicity detection, we usually choose NK cell expansion products with a purity of 85% and higher, to minimize the impact of differences in cytotoxicity caused by high and low NK cell proportions.

*FCM-based killing assay:* eNK cells were labeled with 0.5 μM CFSE (#C34570, ThermoFisher) according to provider’s instruction and subsequently co-cultured with CD20^+^ Daudi cells at an E:T ratio of 5:1. Three experimental groups were established as follows: an ADCC killing group supplemented with 10 µg/mL rituximab (RTX, Roche); a natural killing group without RTX; and a target cell-only group serving as a negative control. After a 6-hour incubation period at 37°C in a 5% CO_2_ incubator, cells were stained with FVD780 and 7-AAD to identify dead cells. Precision Count Beads (Biolegend) were introduced to each sample before FCM analysis, allowing for normalization by acquiring 3,000 beads per sample. The CFSE^–^FVD780^–^7-AAD^–^ subpopulation was gated, and the absolute number and proportion of live cells was determined using FlowJo™ V10.8.1 ^7^.

*eNK cell-mediated killing for tumor spheres:* mCherry-expressing HCT116 cells were seeded in low-adherence U-bottom 96-well plates (ThermoFisher) at a density of 300 cells per well. They were then cultured for 3 days at 37°C in a 5% CO_2_ incubator, allowing them to aggregate and form tumor spheres. Subsequently, eNK cells were labeled with 0.5 μM CellTrace™ Violet (CTV) dye (ThermoFisher), following the manufacturer’s instructions, to enable tracking during the co-culture. The labeled eNK cells were then co-cultured with tumor spheres at E:T ratios of 1:1 and 5:1, with and without 10 µg/mL CET. Following the incubation of 48 hours, we analyzed the size and area of the tumor spheres, as well as the mean expression of mCherry by using a fluorescence microscopy (Leica TCS SP5) and the BioTek Cytation 5 (Agilent) imaging system.

**References**

1. Phan MT, Kim J, Koh SK, et al. Selective Expansion of NKG2C+ Adaptive NK Cells Using K562 Cells Expressing HLA-E. *International journal of molecular sciences*. Aug 20 2022;23(16)doi:10.3390/ijms23169426

2. Coenon L, Rigal E, Courot H, et al. Generation of non-genetically modified, CAR-like, NK cells. *Journal for immunotherapy of cancer*. Jul 18 2024;12(7)doi:10.1136/jitc-2024-009070

3. Sanchez-Martinez D, Allende-Vega N, Orecchioni S, et al. Expansion of allogeneic NK cells with efficient antibody-dependent cell cytotoxicity against multiple tumors. *Theranostics*. 2018;8(14):3856-3869. doi:10.7150/thno.25149

4. Rolle A, Pollmann J, Ewen EM, et al. IL-12-producing monocytes and HLA-E control HCMV-driven NKG2C+ NK cell expansion. *The Journal of clinical investigation*. Dec 2014;124(12):5305-16. doi:10.1172/JCI77440

5. Kleiveland CR. Peripheral Blood Mononuclear Cells. In: Verhoeckx K, Cotter P, Lopez-Exposito I, et al, eds. *The Impact of Food Bioactives on Health: in vitro and ex vivo models*. 2015:161-7.

6. Reina-Ortiz C, Constantinides M, Fayd-Herbe-de-Maudave A, et al. Expanded NK cells from umbilical cord blood and adult peripheral blood combined with daratumumab are effective against tumor cells from multiple myeloma patients. *Oncoimmunology*. Dec 29 2020;10(1):1853314. doi:10.1080/2162402X.2020.1853314

7. Constantinides M, Fayd'herbe De Maudave A, Potier-Cartereau M, Campos-Mora M, Cartron G, Villalba M. Direct Cell Death Induced by CD20 Monoclonal Antibodies on B Cell Lymphoma Cells Revealed by New Protocols of Analysis. *Cancers*. Feb 9 2023;15(4)doi:10.3390/cancers15041109

**Supplemental tables**

**Table S1.** Summary statistics to experiments (CD107a^+^ NK cells) shown in Figure S2.

| **Gating** | | | **% in CD107a^+^ PB-NK subset** | | | | **% in CD107a^+^ UCB-NK subset** | | | |
| --- | --- | --- | --- | --- | --- | --- | --- | --- | --- | --- |
|  | **I** | **T** | **NF** | **K562** | **PLH** | **221.AEH** | **NF** | **K562** | **PLH** | **221.AEH** |
| G1: | + | – | 2.13±1.37 | 6.83±3.34 | 3.38±1.81 | 4.30±2.61 | 25.9±6.92 | 7.08±4.00 | 3.40±2.95 | 6.94±6.73 |
| G2: | + | + | 3.93±1.46 | 21.0±8.36 | 7.34±2.25 | 10.5±4.27 | 11.6±11.2 | 62.2±14.2 | 65.6±19.6 | 59.5±18.0 |
| G3: | – | + | 11.6±2.31 | 14.8±1.38 | 17.4±5.05 | 22.0±6.21 | 9.89±3.36 | 16.5±2.74 | 17.0±5.55 | 17.4±4.83 |
| G4: | – | – | 82.4±1.86 | 57.4±11.9 | 71.9±8.93 | 63.2±11.8 | 52.5±14.4 | 14.2±9.78 | 14.1±14.4 | 16.2±11.5 |

Notes: Analysis of cytokines production after co-culturing CD56^+^CD3^–^ NK cells with K562, PLH, or 221.AEH cells for 6 hours. The negative control group, denoted as “no feeder cells (NF)” was included. The mean percentage of cytokines production was assessed by FCM. The sum of cell proportions from gating G1 to G4 is approximately 100%. Sample numbers: PB-NK, n=4; UCB-NK, n=5. ± represent SD. Abbreviations: PB, peripheral blood; UCB, umbilical cord blood; NK, natural killer; I, IFN-γ; T, TNF-α; FCM, flow cytometry; G, group; SD, standard deviation.

**Table S2.** Summary statistics to experiments (CD107a^–^ NK cells) shown in Figure S2.

| **Gating** | | | **% in CD107a^–^ PB-NK subset** | | | | **% in CD107a^–^ UCB-NK subset** | | | |
| --- | --- | --- | --- | --- | --- | --- | --- | --- | --- | --- |
|  | **I** | **T** | **NF** | **K562** | **PLH** | **221.AEH** | **NS** | **K562** | **PLH** | **221.AEH** |
| G5: | + | – | 0.17±0.11 | 1.00±0.38 | 0.64±0.29 | 1.28±0.80 | 0.22±0.17 | 4.44±1.09 | 1.03±0.36 | 1.88±1.09 |
| G6: | + | + | 0.13±0.10 | 1.78±0.83 | 0.97±0.52 | 2.50±1.80 | 0.14±0.14 | 25.6±11.3 | 4.40±2.32 | 5.01±2.01 |
| G7: | – | + | 0.75±0.28 | 3.98±1.54 | 3.61±1.23 | 6.34±2.71 | 0.80±0.80 | 17.0±5.67 | 10.5±4.81 | 11.2±3.87 |
| G8: | – | – | 99.0±0.37 | 93.3±2.72 | 94.8±2.02 | 90.0±5.24 | 98.8±1.02 | 53.0±15.8 | 84.0±7.16 | 81.9±5.61 |

Notes: Analysis of cytokines production after co-culturing CD56^+^CD3^–^ NK cells with K562, PLH, or 221.AEH cells for 6 hours. The negative control group, denoted as “no feeder cells (NF)” was included. The mean percentage of cytokines production was assessed by FCM. The sum of cell proportions from gating G5 to G8 is approximately 100%. Sample numbers: PB-NK, n=4; UCB-NK, n=5. ± represent SD. Abbreviations: PB, peripheral blood; UCB, umbilical cord blood; NK, natural killer; I, IFN-γ; T, TNF-α; FCM, flow cytometry; G, group; SD, standard deviation.

**Supplemental figures**


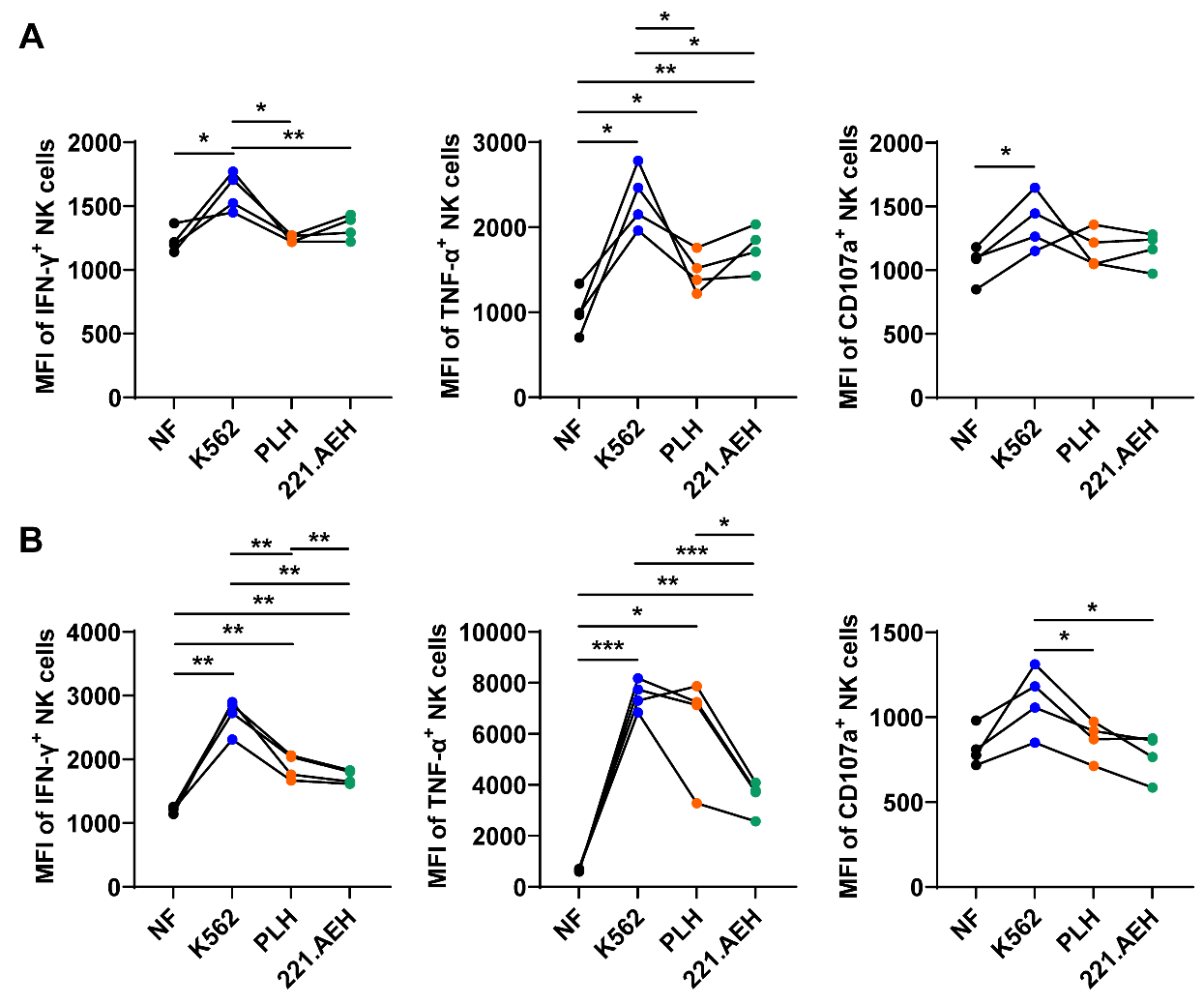


**Figure S1. Expression of MFI in IFN-γ^+^, TNF-α^+^, and CD107a^+^ cells after stimulation of PB-NK and UCB-NK cells with feeder cells.** After co-culturing PB-NK (A) and UCB-NK (B) cells with three types of feeder cells (K562, PLH, and 221.AEH) for 6 hours, line graphs showing the expression of corresponding MFI in IFN-γ^+^, TNF-α^+^, and CD107a^+^ cells (PB-NK, n=4; UCB-NK, n=4). The NF group served as the baseline expression. Dots linked by a line represent data collected from the same donor. Two-tailed paired t-tests were used for all comparisons. *p < 0.05; **p < 0.01; ***p < 0.001.

**
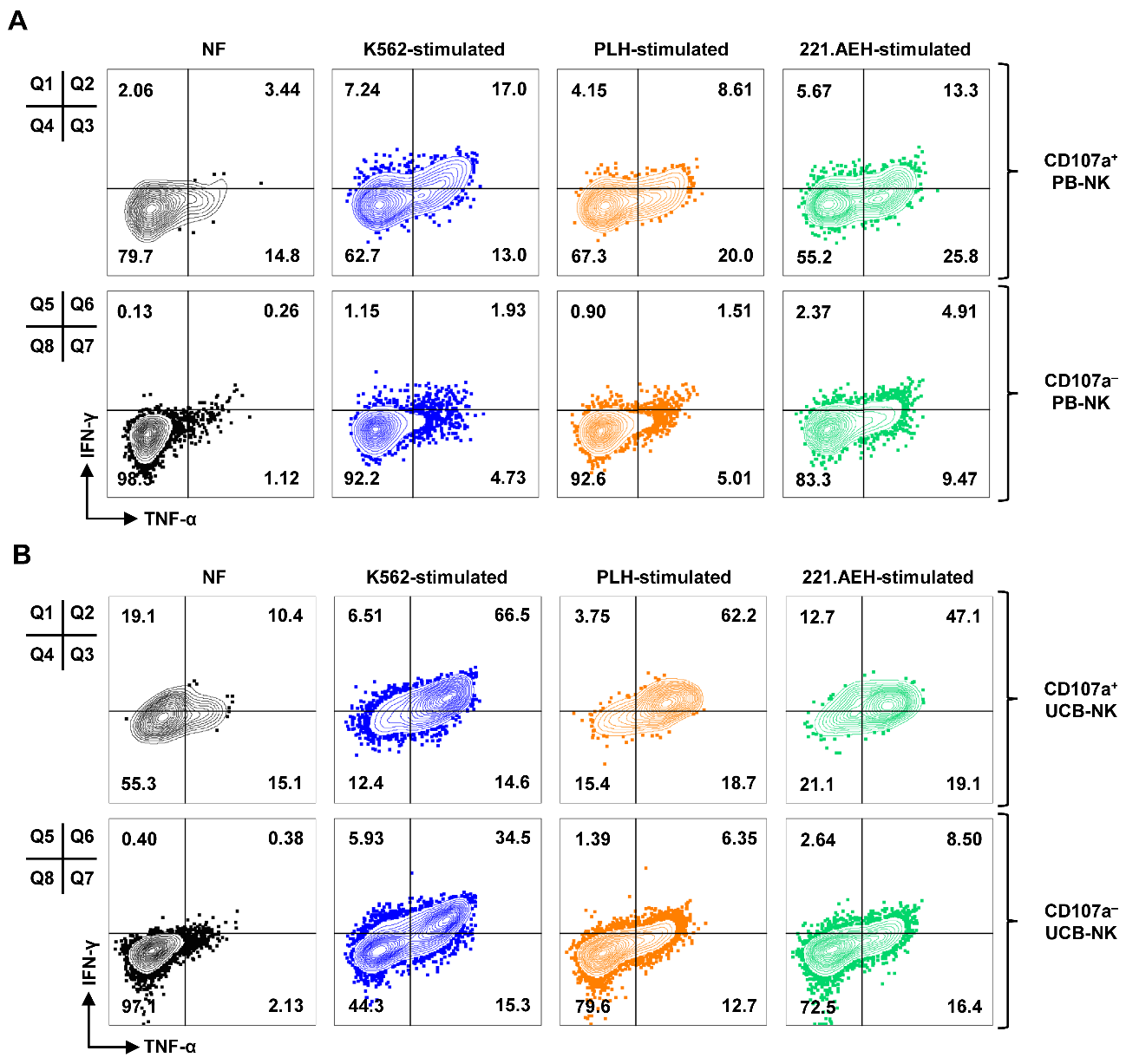
Figure S2. Assessment of the capacity of CD107a^+^ and CD107a^–^ NK cells to produce IFN-γ and TNF-α after stimulation with feeder cells.** Representative FCM contour plots illustrating the proportion of IFN-γ^+^ and TNF-α^+^ in CD107a^+^ and CD107^–^ subpopulations from PB-NK (A) and UCB-NK (B) cells after 6 hours of stimulation with K562, PLH, and 221.AEH cells. The NF group served as the negative control.


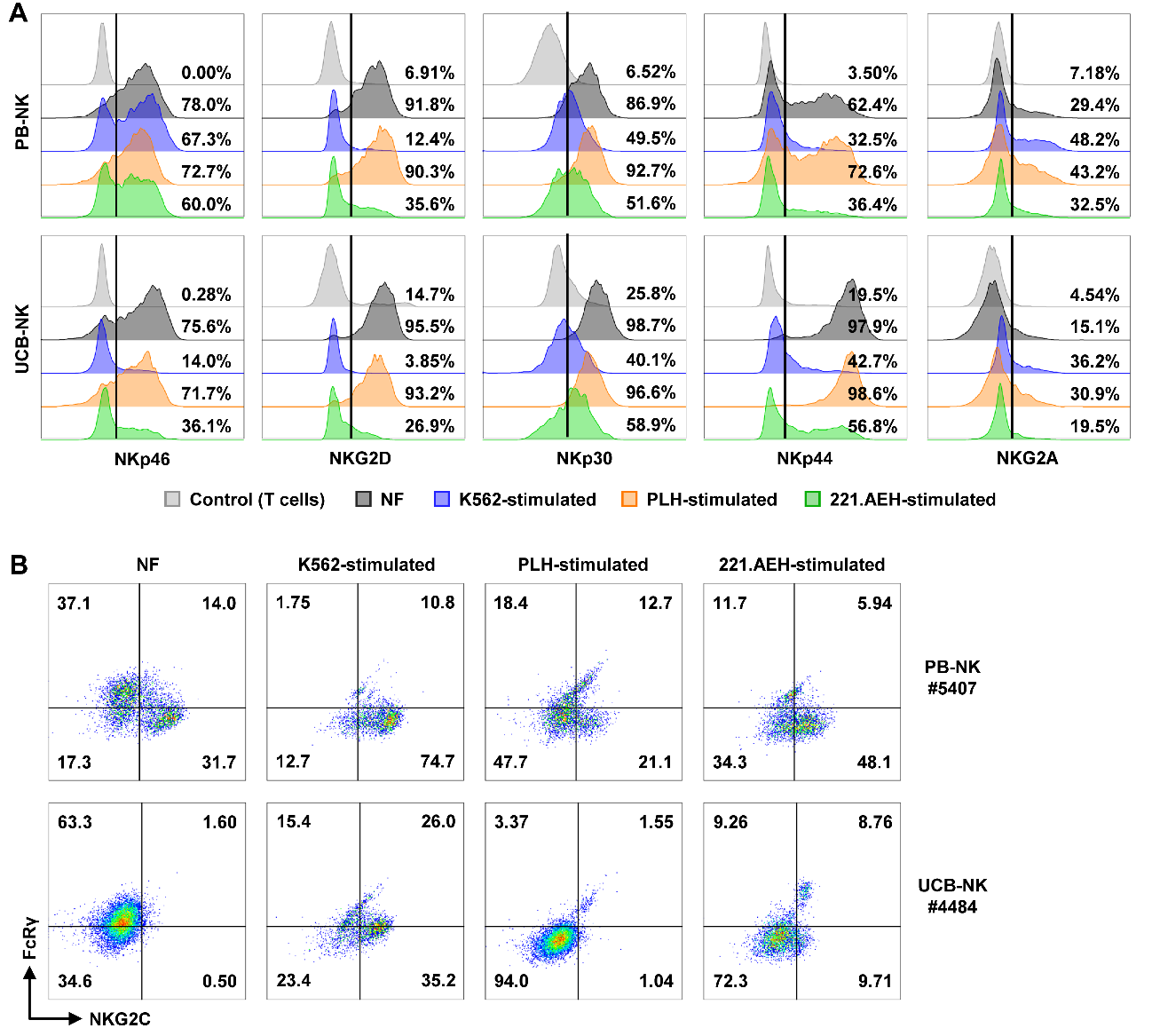


**Figure S3. Changes in NK cell phenotypes after encountering feeder cells.** (A) The expression levels of NKp46, NKG2D, NKp30, NKp44, and NKG2A on PB-NK and UCB-NK cells were determined by FCM after 3 days stimulation with K562, PLH, and 221.AEH cells. Representative FCM histograms are shown here. (B) After 3 days of co-culture with K562, PLH and 221.AEH cells, the proportion of FcRγ^–^ and NKG2C^+/–^ subsets in PB-NK and UCB-NK cells was analyzed using FCM. Representative FCM dot plots are presented here. The NF group served as the negative control.


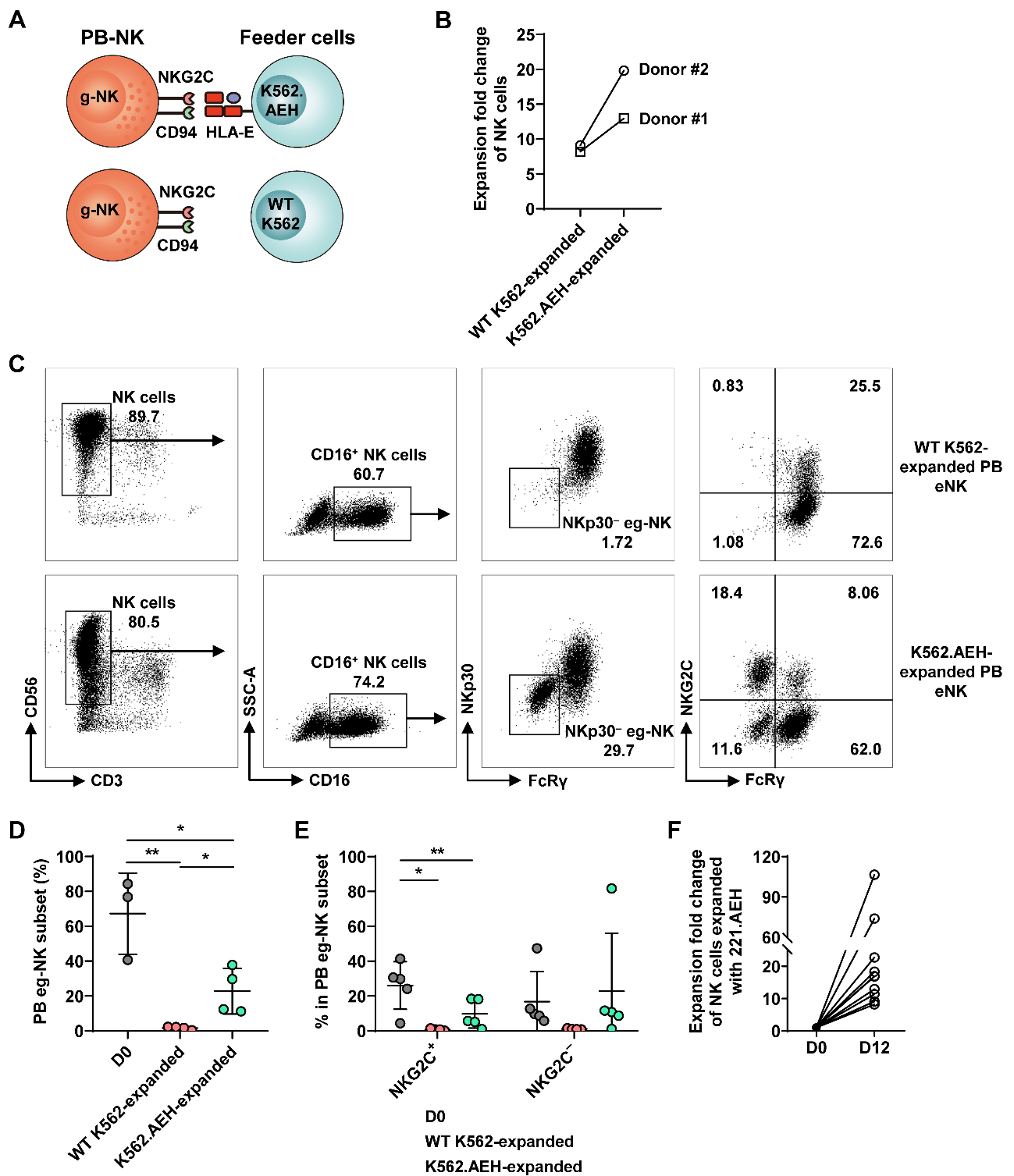


**Figure S4. Expansion of PB-NK cells with HLA-E overexpressing feeder cells.** (A) A schematic representation of g-NK cell expansion in PB-NK cells with K562.AEH and WT K562 feeder cells. (B) Comparison of the expansion efficiency of PB eNK cells expanded by K562.AEH and WT K562 feeder cells after 12 days (n=2). (C) Representative FCM dot plots showing phenotypic differences in PB eNK cells expanded with the two feeder cell types, particularly the g-NK cell-related phenotype. (D and E) Scatter plot graphs presenting changes in the proportion of NKp30^–^ PB eg-NK subset pre- and post-expansion, as well as NKG2C^+/–^ PB eg-NK subsets. (D) D0 group: n=3; WT K562-expanded group: n=4; K562.AEH-expanded group: n=4. (E) D0 group: n=5; WT K562-expanded group: n=4; K562.AEH-expanded group: n=5. (F) Fold expansion of 221.AEH-expanded PB eNK cells after 12 days of expansion (n=10). D0 indicates the beginning of expansion. Dots linked by a line represent data collected from the same donor. Error bars represent SD. Two-tailed unpaired t-tests were used for all comparisons. *p < 0.05; **p < 0.01.


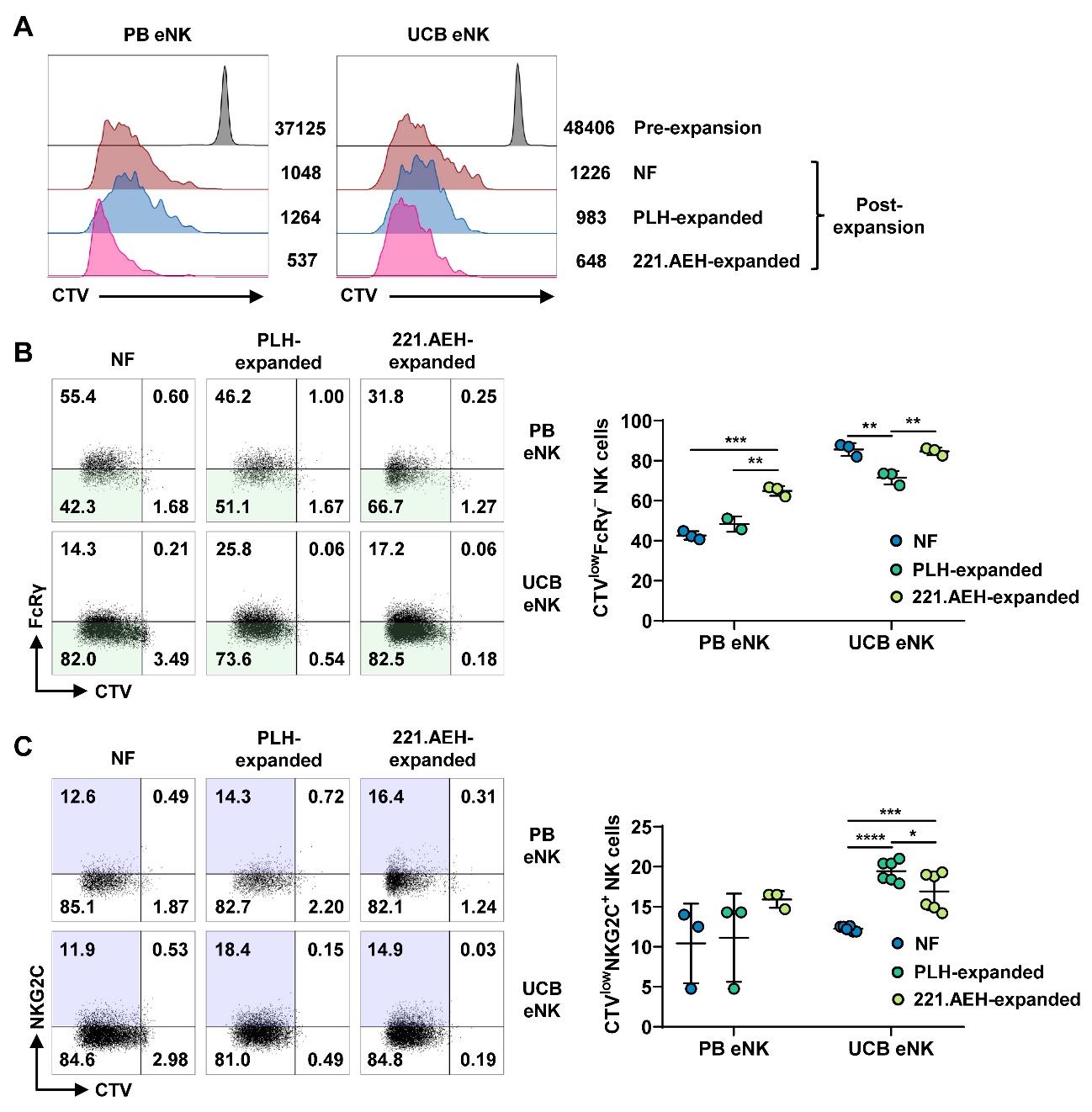


**Figure S5. Proliferation of PB eNK and UCB eNK cells.** (A) Following 7 days of stimulation with PLH or 221.AEH cells, supplemented with 100 IU/mL IL-2 and 5 ng/mL IL-15, 0.5 μM CTV-labeled PB eNK and UCB eNK cells were analyzed using FCM to assess changes in the CTV proliferation peaks. Representative FCM histograms are shown here. (B and C) Expression of FcRγ (B) and NKG2C (C) in proliferating PB eNK and UCB eNK cells upon stimulation with PLH and 221.AEH cells. The NF group represents the addition of only IL-2 and IL-15 cytokines into the culture system. Representative FCM dot plots are presented here. (B) PB eNK: NF group, n=3; PLH-expanded group, n=2; 221.AEH-expanded group, n=3. UCB eNK: NF group, n=3; PLH-expanded group, n=3; 221.AEH-expanded group, n=3. (C) PB eNK: NF group, n=3; PLH-expanded group, n=3; 221.AEH-expanded group, n=3. UCB eNK: NF group, n=6; PLH-expanded group, n=6; 221.AEH-expanded group, n=6. Error bars indicate SD. Two-tailed unpaired t-tests were used for all comparisons. *p < 0.05, **p < 0.01, ***p < 0.001, ****p < 0.0001.


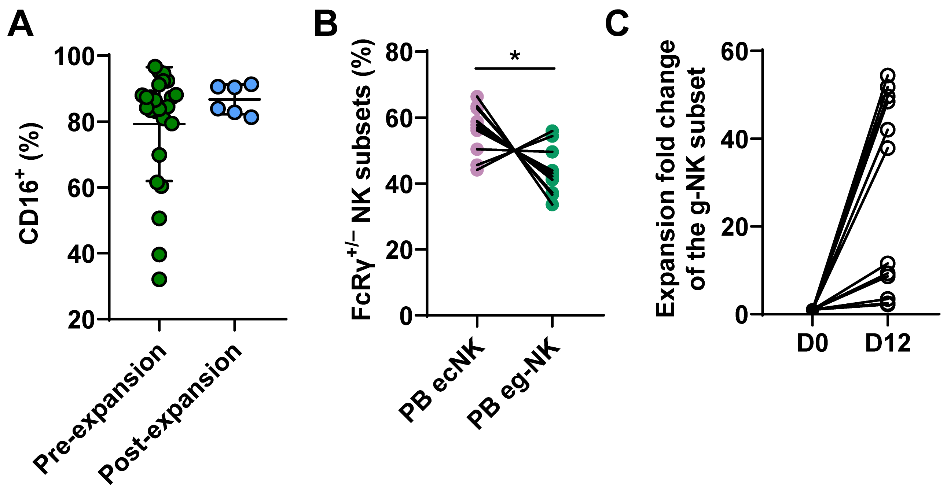


**Figure S6.** **Characteristics of PB eNK cells expanded with 221.AEH feeder cells.** (A) Scatter plot graph showing the expression of CD16 on CD45^+^CD3^–^CD19^–^CD56^+^ NK cells pre- and post-expansion (pre-expansion: n=25; post-expansion: n=6). (B) Comparison of the average percentage of ecNK and eg-NK cells among PB eNK cells (n=11). (C) After 12 days of expanding PB-NK cells using the 221.AEH feeder cells, the expansion efficiency of the PB eg-NK subset was compared to the initial expansion stage (n=12). Dots linked by a line represent data collected from the same donor. Error bars indicate SD. Two-tailed unpaired and paired t-tests were used for all comparisons. *p < 0.05.


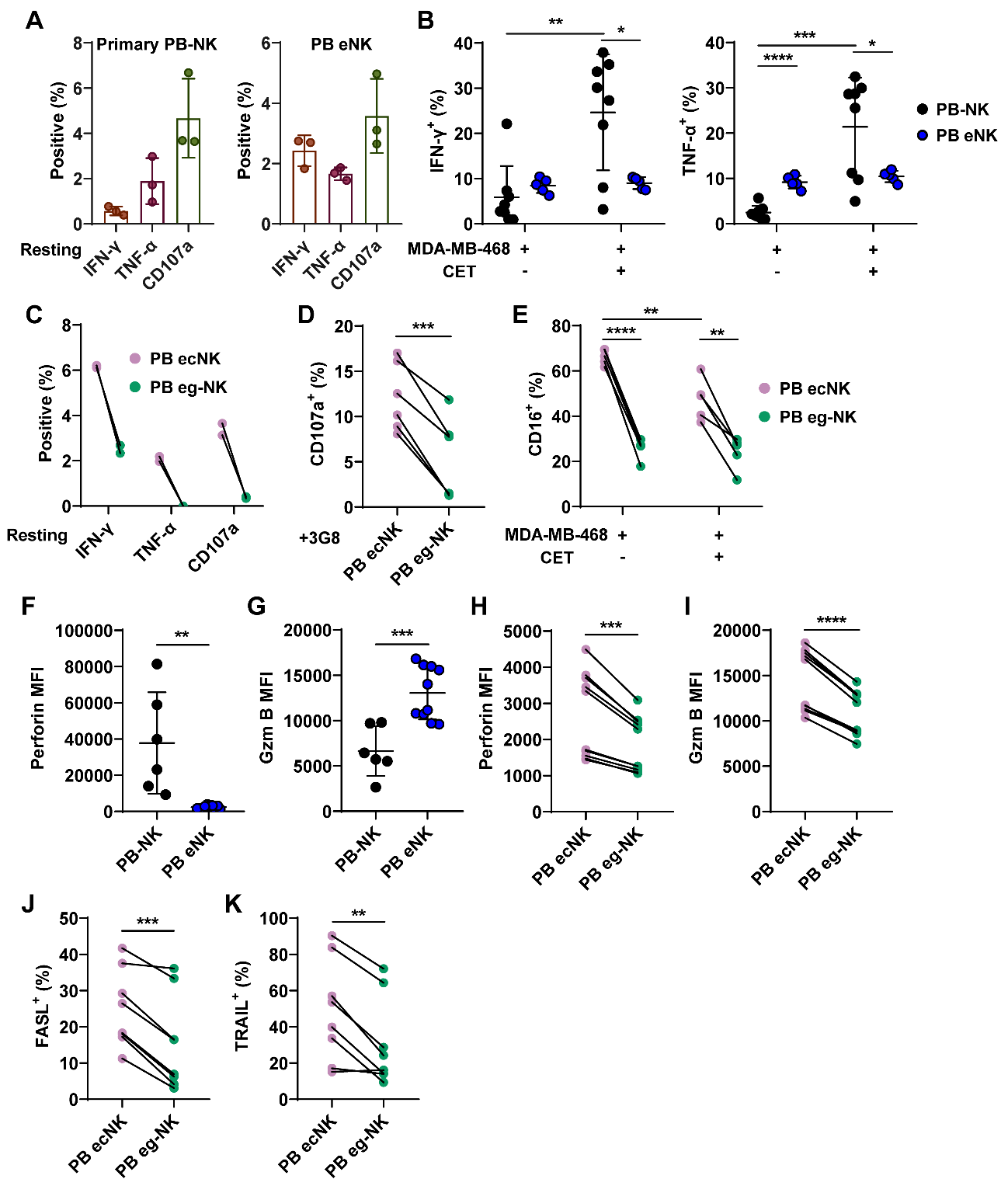


**Figure S7. Functional analysis of PB eNK cells.** (A) In the absence of stimulation, using FCM to assess the baseline levels of IFN-γ and TNF-α production, as well as CD107a expression, in primary PB-NK (n=3) and PB eNK cells (n=3). (B) To assess the production capacity of IFN-γ and TNF-α, PB-NK and PB eNK cells were stimulated for 6 hours with MDA-MB-468 cells opsonized with 2 µg/mL CET or without CET (PB-NK, n=8; PB eNK, n=5). (C) Proportion of IFN-γ^+^, TNF-α^+^, and CD107a^+^ in PB ecNK and PB eg-NK cells under the resting state (n=2). (D) Comparison of the degranulation capacity of PB ecNK and PB eg-NK cells after 6 hours of stimulation with 2 µg/mL immobilized 3G8 (n=6). (E) Expression of CD16 on PB ecNK and PB eg-NK cells upon stimulation as above described (n=5). (F and G) Comparison of perforin (F) and Gzm B (G) expression in PB-NK and PB eNK cells. (F) Perforin: PB-NK, n=6; PB eNK, n=10. (G) Gzm B: PB-NK, n=6; PB eNK, n=10. (H-K) Comparison of cytotoxicity-mediating molecules expression in UCB ecNK and UCB eg-NK cells. (H) Perforin: n=10. (I) Gzm B: n=10. (J) FASL: n=8. (K) TRAIL: n=8. Dots linked by a line represent data collected from the same donor. Error bars indicate SD. Two-tailed unpaired and paired t-tests were used for all comparisons. *p < 0.05; **p < 0.01; ***p < 0.001; ****p < 0.0001.


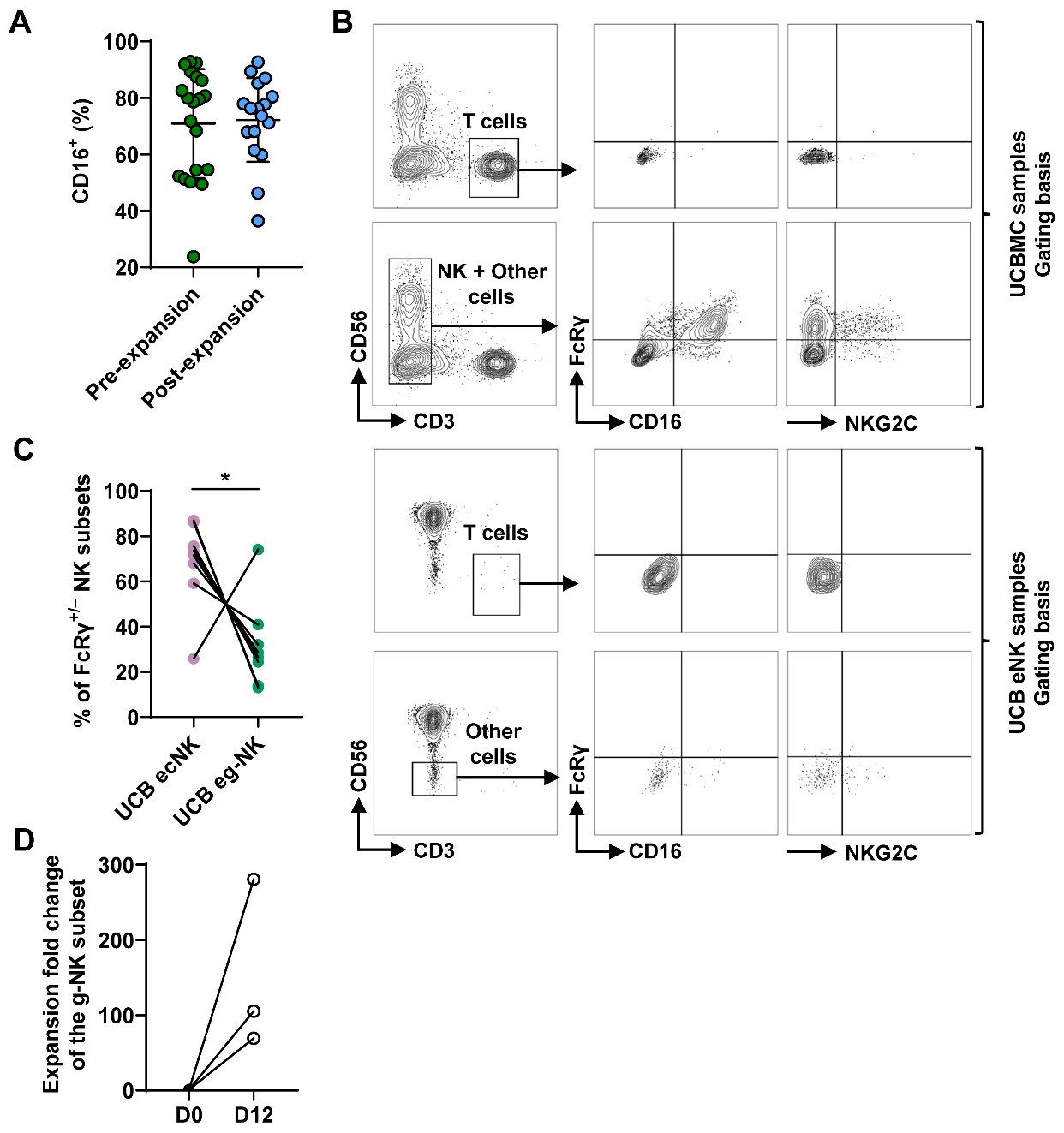


**Figure S8. Characteristics of UCB eNK cells expanded with PLH feeder cells.** (A) Scatter plot graph showing the expression of CD16 on CD45^+^CD3^–^CD19^–^CD56^+^ NK cells pre- and post-expansion (pre-expansion group: n=20; post-expansion group: n=17). (B) Gating strategy used for defining the cNK and g-NK subsets within UCB-NK and UCB eNK cells. T cells are FcRγ-negative, while some of the other populations partially express FcRγ. (C) Before-after plot graph showing average percentage of ecNK and eg-NK cells among UCB eNK cells (n=9). (D) After 12 days of expanding UCB-NK cells using the PLH expansion system, the expansion effect of the UCB eg-NK subset was compared to the initial expansion stage (n=3). Dots linked by a line represent data collected from the same donor. Error bars indicate SD. Two-tailed unpaired and paired t-tests were used for all comparisons. *p < 0.05.


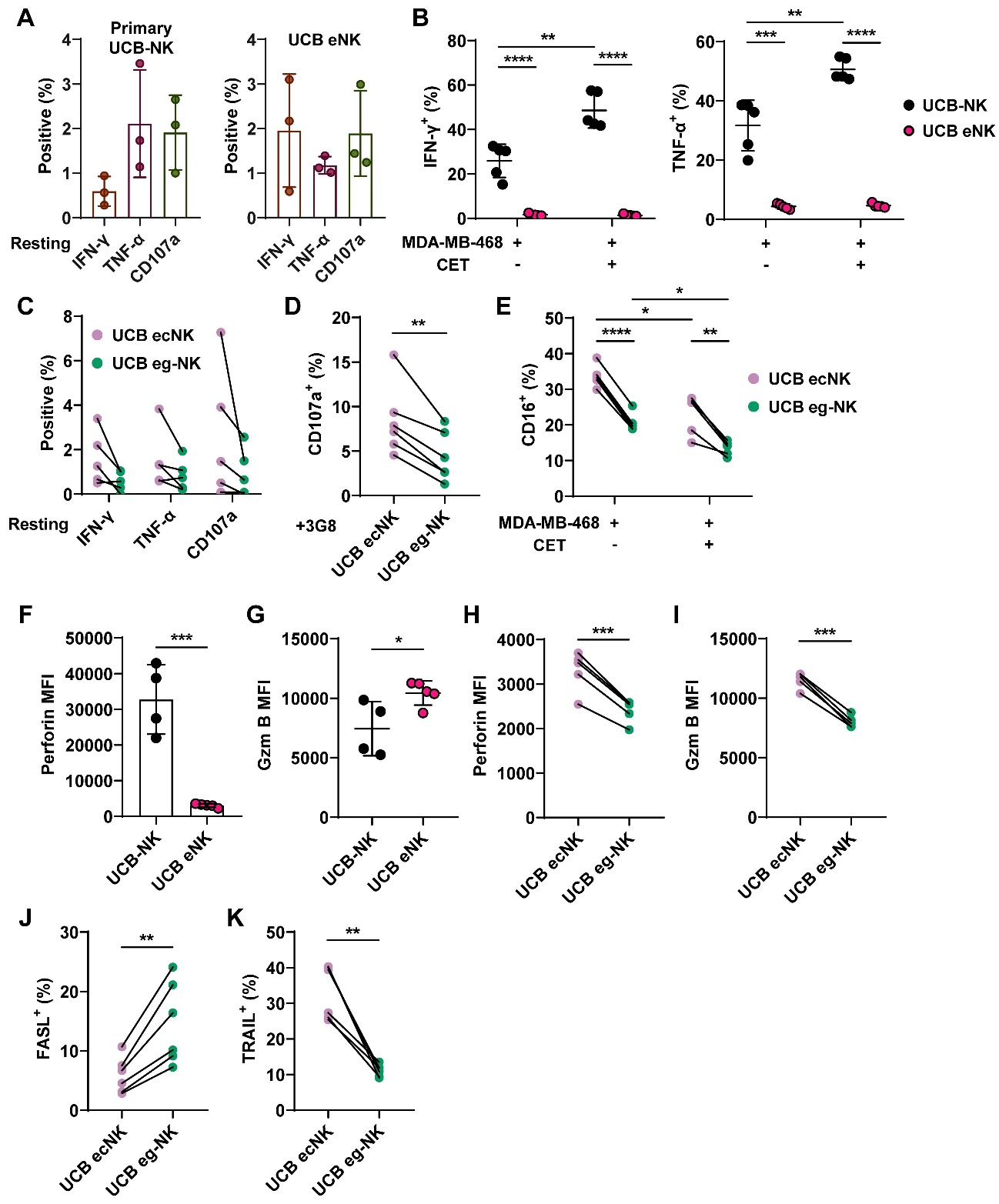


**Figure S9. Functional analysis of UCB eNK cells.** (A) Without external stimulation, FCM was employed to evaluate the baseline levels of IFN-γ and TNF-α production, as well as CD107a expression, in primary UCB-NK (n=3) and UCB eNK (n=3) cells. (B) Comparison of cytokines production (IFN-γ and TNF-α) in UCB-NK and UCB eNK cells pre- and post-expansion, upon stimulation with MDA-MB-468 cells in the presence or absence of 2 µg/mL CET (UCB-NK, n=5; UCB eNK, n=5). (C) The proportion of IFN-γ^+^, TNF-α^+^, and CD107a^+^ in resting UCB ecNK and UCB eg-NK cells was determined using FCM (n=5). (D) Evaluation of CD107a expression in UCB ecNK and UCB eg-NK cells following 6-hour stimulation with 2 µg/mL immobilized 3G8 (n=6). (E) Expression of CD16 on UCB ecNK and UCB eg-NK cells upon stimulation as above described (n=5). (F and G) Comparison of perforin (F) and Gzm B (G) expression in UCB-NK and UCB eNK cells. (F) Perforin: UCB-NK, n=4; UCB eNK, n=5. (G) Gzm B: UCB-NK, n=4; UCB eNK, n=5. (H-K) Comparison of cytotoxicity-mediating molecules expression in UCB ecNK and UCB eg-NK cells. (H) Perforin: n=5. (I) Gzm B: n=5. (J) FASL: n=6. (K) TRAIL: n=6. Dots linked by a line represent data collected from the same donor. Error bars indicate SD. Two-tailed unpaired and paired t-tests were used for all comparisons. *p < 0.05; **p < 0.01; ***p < 0.001; ****p < 0.0001.


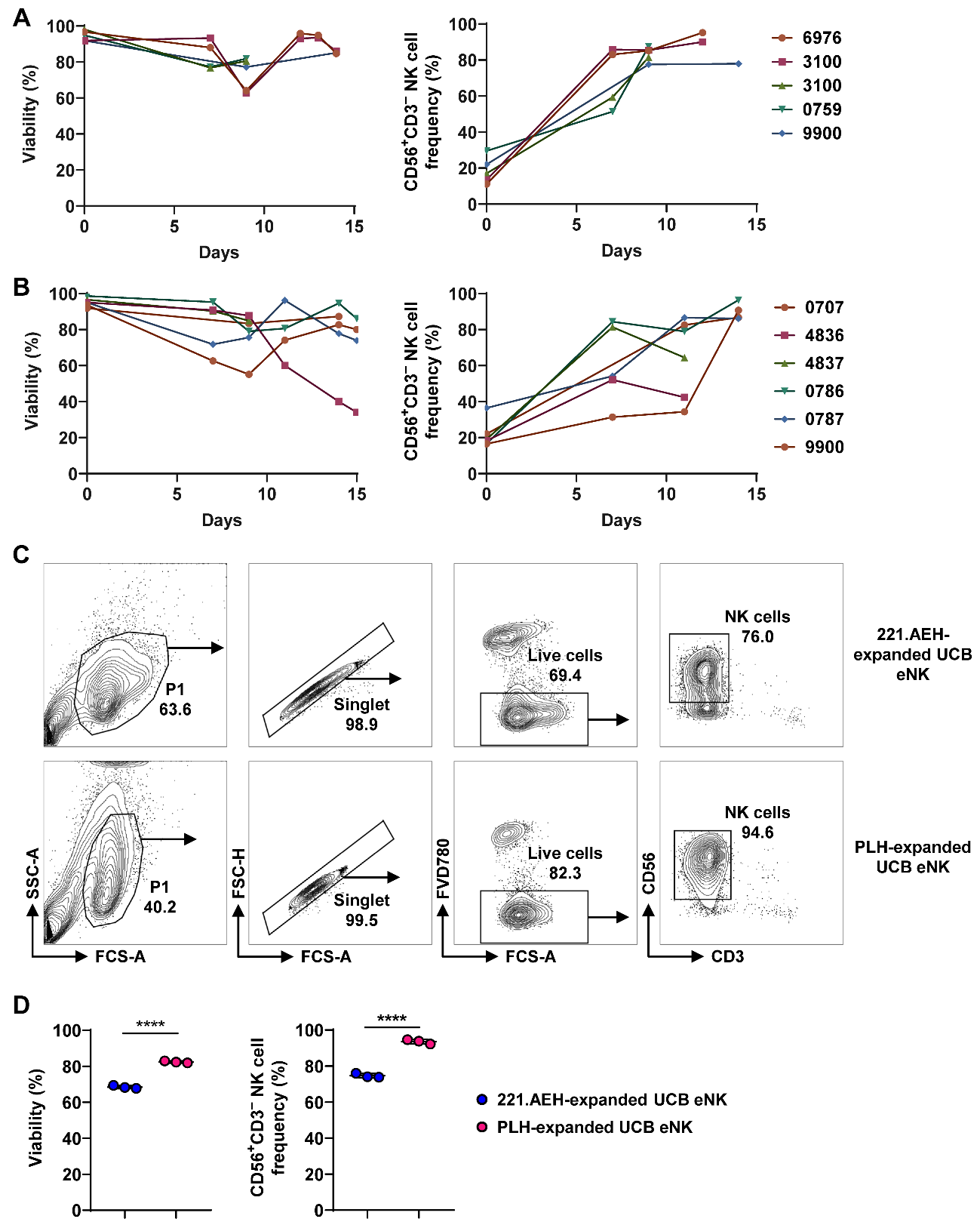


**Figure S10.** **Comparison of eNK cells expanded with 221.AEH and PLH feeder cells.** (A and B) Several donor-derived PB-NK cells were expanded with 221.AEH (A) and PLH (B) feeder cells for 15 days. Muse^®^ Cell Analyzer and FCM were used to evaluate the viability of PB eNK cells and the CD56^+^CD3^–^ NK cell frequency. 221.AEH as feeder cell group: n=5; PLH as feeder cell group: n=6. (C) Representative FCM contour plots demonstrating cell viability and the proportion of CD56^+^CD3^−^ NK cells after expansion of UCB-NK cells using 221.AEH and PLH feeder cells. (D) Comparison of cell viability and NK cell purity of UCB eNK cells expanded by the two expansion systems (n=3). Dots linked by a line represent data collected from the same donor. Error bars indicate SD. Two-tailed unpaired t-tests were used for all comparisons. ****p < 0.0001.


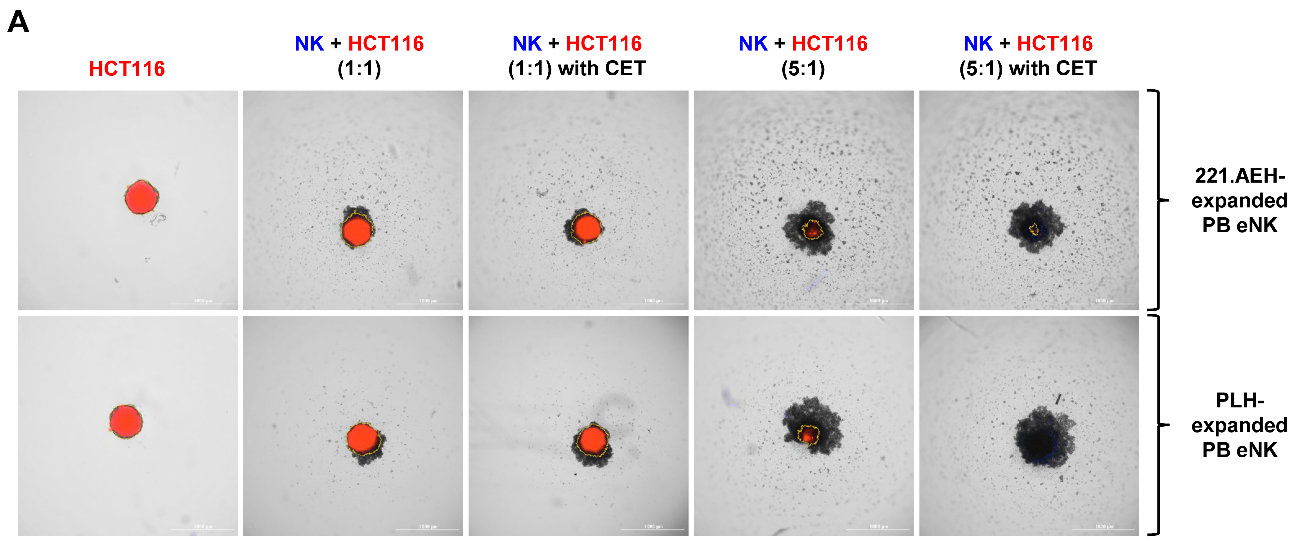


**Figure S11. Assessment of cytotoxicity in HCT116 tumor spheres targeted by 221.AEH- or PLH-expanded PB eNK cells.** (A) Red fluorescent spheres represent mCherry^+^ HCT116 tumor cells, while blue fluorescence represents CTV-labeled eNK cells. After co-culturing PB eNK cells with HCT116 tumor spheres at ratios of 1:1 and 5:1 for 48 hours, in the presence or absence of 10 µg/mL CET, data were acquired using Cytation Imaging Multi-Mode Readers. A smaller area and size of red fluorescent sphere indicate lower survival of tumor cells.
